# CRISPR/Cas9 editing reveals novel mechanisms of clustered microRNA regulation and function

**DOI:** 10.1038/s41598-017-09268-0

**Published:** 2017-08-17

**Authors:** Lazaros Lataniotis, Andreas Albrecht, Fatma O. Kok, Clinton A. L. Monfries, Lorena Benedetti, Nathan D. Lawson, Simon M. Hughes, Kathleen Steinhofel, Manuel Mayr, Anna Zampetaki

**Affiliations:** 1 King’s British Heart Foundation Centre, King’s College London, London, UK; 2 Middlesex University, School of Science and Technology, London, UK; 3 University of Massachusetts Medical School, Department of Molecular, Cell, and Cancer Biology, Worcester, USA; 4 King’s College London, Randall Division, London, UK; 5 King’s College London, Division of Cancer Studies, London, UK; 6 King’s College London, Informatics Department, London, UK

## Abstract

MicroRNAs (miRNAs) are important regulators of diverse physiological and pathophysiological processes. MiRNA families and clusters are two key features in miRNA biology. Here we explore the use of CRISPR/Cas9 as a powerful tool to delineate the function and regulation of miRNA families and clusters. We focused on four miRNA clusters composed of miRNA members of the same family, homo-clusters or different families, hetero-clusters. Our results highlight different regulatory mechanisms in miRNA cluster expression. In the case of the miR-497~195 cluster, editing of miR-195 led to a significant decrease in the expression of the other miRNA in the cluster, miR-497a. Although no gene editing was detected in the miR-497a genomic locus, computational simulation revealed alteration in the three dimensional structure of the pri-miR-497~195 that may affect its processing. In cluster miR-143~145 our results imply a feed-forward regulation, although structural changes cannot be ruled out. Furthermore, in the miR-17~92 and miR-106~25 clusters no interdependency in miRNA expression was observed. Our findings suggest that CRISPR/Cas9 is a powerful gene editing tool that can uncover novel mechanisms of clustered miRNA regulation and function.

## Introduction

MicroRNAs (miRNAs) are small non coding RNAs that control gene expression posttranscriptionally^1^. In the cardiovascular diseases and in cancer, miRNAs have emerged as prominent regulators in disease initiation and progression. Circulating miRNA signatures were also shown to associate with cardiovascular risk and were proposed as a novel mechanism of intercellular communication^2–4^. Several of the identified miRNAs with key functions in the cellular responses are organized in miRNA clusters^5^. These consist of multiple miRNAs located at the same chromosomal locus that are transcribed as a single primary miRNA transcript. The formation of structural clusters was proposed to enhance stability by preventing immediate degradation and ensuring biogenesis of miRNAs that regulate a similar set of genes thus having direct functional implications^6^. In many cases, clustered miRNAs belong to the same miRNA family^7, 8^ forming a homo-cluster. Such miRNAs have identical ‘seed region’ and share a high degree of sequence homology that leads to functional redundancy^9, 10^ but also poses a serious limitation in miRNA research^11^.

CRISPR/Cas9 is an RNA-guided gene editing platform that is simple to design, highly specific and easy to use^12, 13^. It consists of a sole nuclease (Cas9) that identifies a conserved three nucleotide proto-adjacent motif (PAM) and cleaves both DNA strands thus creating a double stranded break (DSB). The CRISPR RNA (crRNA) is a short RNA with variable sequence that in combination with an adaptor trans-activating RNA (tracrRNA) acts as a guide for Cas9. The crRNA and tracrRNA can be fused to create the single-guide RNA (sgRNA)^14^. This is a highly flexible and easy to design short RNA molecule that can direct Cas9 to any target in the immediate vicinity of the PAM sequence by altering only the 20-nt guide sequence within the sgRNA. Once in complex, the Cas9-sgRNA will interrogate the DNA. Upon binding to the PAM, the Cas9-sgRNA complex detects DNA complementarity to the guide RNA and cleaves each DNA strand to generate a blunt DSB, usually, at a position three base pairs from the PAM^15, 16^. Initial reports demonstrated the feasibility of using the CRISPR/Cas9 platform in targeting miRNA expression albeit inducing a varying degree of inhibition^17, 18^. Further studies improved the efficiency^19–21^. *In vivo*, the CRISPR/Cas9 platform was used to delete a 53-kb fragment and generate knockout mice to elucidate the function of the largest miRNAs cluster in mice^22^, while the first miRNA double knockout mice using the CRISPR/Cas9 system have also been reported^23^. Recently, a robust approach to establish a multiplex system to achieve targeting of entire miRNA families was developed^24^. CRISPR/Cas9 technology also emerged as a powerful platform to identify new regulators of the miRNA pathway^25^ and RNA binding proteins involved in miRNA biogenesis^26^.

In the present study we explore the use of gene editing to delineate clustered miRNA regulation. To this end we employed the CRISPR/Cas9 system and determined the effect of gene editing in four miRNA clusters composed of miRNAs belonging to the same or distinct families. Our findings indicate that CRISPR/Cas9 is a powerful technology that can provide novel insights into the structural constraints and mechanisms regulating the expression of clustered miRNAs.

## Materials and Methods

### Cell culture

Vascular Smooth muscle cells (VSMCs) were isolated by enzymatic digestion of mouse aortas, as described elsewhere^27^ and cultured in Dulbecco’s Modified Eagle’s Medium (DMEM, ThermoFisher Scientific, Runcorn, UK) supplemented with 10% heat-inactivated Foetal Bovine Serum (FBS), 2 mM L-glutamine and 1% penicillin/streptomycin (100 U/ml penicillin and 100 µg/ml streptomycin), at 37 °C in a humidified atmosphere of 95% air/5% CO_2_. VSMCs were plated on gelatin coated flasks (0.04% in DMEM, ThermoFisher Scientific, Runcorn, UK) and subcultured with a ratio of 1:3 every 3 days. Human Embryonic Kidney (HEK) 293 T cells (HEK 293 T) were obtained from ATCC (CRL-3216). The cells were cultured in DMEM, supplemented with 10% FBS, 2 mM L-glutamine and 1% penicillin/streptomycin (100 U/ml penicillin and 100 µg/ml streptomycin) and subcultured with a ratio of 1:3 or 1:4 every 2–3 days^28^.

All methods were carried out in accordance with the institutional guidelines and regulations. All experimental protocols were approved by the KCL licensing committee.

### Single Guide RNA Design and *In Vitro* Transcription (IVT)

The online guide design tool (http://crispr.mit.edu) was used to identify sgRNAs. The DNA sequence corresponding to the transcript annotated in miRBase v21 as stem loop miRNA was used as input sequence to design sgRNAs (Supplementary Table 1). The highest scoring guides, which targeted sequences either in or close to the miRNA stem-loop, were selected. *In vitro* transcription (IVT) was performed using the GeneArt Precision sgRNA Synthesis Kit (ThermoFisher Scientific, Runcorn, UK) according to manufacturer’s recommendations. The DNA template of the sgRNA was PCR assembled and used to generate the sgRNAs by IVT. All IVT target primer sets and PCR primers are provided in Supplementary Table 2.

### Lentiviral particle transduction

To confer Cas9 expression in VSMCs we used the LentiCRISPRv2 vector (Addgene, #52961) that encodes a *Streptococcus pyogenes* Cas9 (SpCas9) under the control of an elongation 1a short promoter (EFS)^29^. Lentiviral particles were produced using the lentiviral vector LentiCRISPRv2 and packaging plasmids pMD2.G and psPAX2 (Addgene, #12259, #12260) as described previously^30^. Transfection complexes were formed using Viafect (Promega). Following a 6 h incubation, the medium was replaced with 8 ml of DMEM with 10% FBS and antibiotics supplemented with 1% Bovine Serum Albumin (Sigma-Aldrich). After 60 h, the lentiviral supernatant was collected, centrifuged at 1000 rpm for 5 min and filtered through 0.45 µm VWR Syringe Filters. A p24 antigen ELISA (Cell Biolabs) was used to determine the viral titer^31^.

### Lentiviral Infection

VSMCs were seeded at a density of 200.000 cells per T25 flask. The following day cells were infected with lentiviral particles (1.6–2.4 × 10^7^ TU/ml)^31^. The media were supplemented with 8 μg/ml of Polybrene (Sigma-Aldrich) and cells were incubated for 24 h with the viral supernatant. Fresh media was then added to the cells. Infected cells were puromycin selected (2 μg/ml from ThermoFisher Scientific, Runcorn, UK) for 48 h^32^.

### Genome editing of VSMCs

To induce gene editing, VSMCs expressing Cas9 were transfected with IVT sgRNA using LipofectamineRNAiMAX (ThermoFisher Scientific, Runcorn, UK) according to the company’s recommendations. In brief, VSMCs were seeded in 6 well plates at a cell density of 150.000 cells/well in complete medium with no antibiotics. The following day, cells were transfected with 65 pmole of IVT sgRNA^33^. Equimolar concentration of a tracrRNA was transfected in control cells. After 24 hours, fresh media were added to the cells and two days later the editing efficiency was determined.

### Genomic PCR and Sanger Sequencing

Genomic DNA extraction was performed using QuickExtract DNA Extract Solution (Epicentre) according to manufacturer’s recommendations. Briefly, cells were lysed in 200 µl of the extraction solution and incubated at 65 °C for 20 min. Subsequently, samples were heated at 96 °C for 10 min and stored at −20 °C. The genomic loci of interest were amplified by PCR using Kapa HiFi PCR kit Hot start (Kapa Biosystems). The PCR amplicons were then column purified (ThermoFisher Scientific, Runcorn, UK). Genomic PCR primer sequences are provided in Supplementary Table 2. To determine the mutation pattern, purified PCR amplicons were cloned into the pGEM-T Vector (Promega). Random colonies were PCR screened and submitted for Sanger sequencing using the T7 primer (TAATACGACTCACTATAGGG).

### T7 Endonuclease I Assay

To assess the gene editing efficiency, the T7 Endonuclease I assay (T7EI) was employed as described recently^34^. Briefly, 90 ng of the purified PCR product in 1x KAPA HiFi GC Buffer were used for the reannealing reaction. PCR products were denatured by heating to 95 °C for 10 min and then re-annealed by slowly ramping down the temperature to 25 °C by a rate of 4.5% per min. Subsequently, samples were supplemented with 0.5 U of T7EI (NEB, Hitchin, UK) and incubated at 37 °C for 1 h. As a final step, one volume of Purple Loading Buffer (NEB, Hitchin, UK) was added and the reaction was quenched by heating to 70 °C for 10 min. To visualize the T7EI digestion products, a 2% agarose gel in 1xTBE buffer was prepared and stained with SYBR Gold Nucleic Acid Gel Stain. The gel was visualized using the Ettan DIGE Imager by GE HealthCare. The T7EI results were quantified using the ImageJ software. The editing efficiency was calculated using the following formula^28^:

$${\rm{Indel}}\,( \% )=(1-{(1-({\rm{B}}+{\rm{C}}/{\rm{A}}+{\rm{B}}+{\rm{C}}))}^{1/2})\times 100,$$

where, A = uncut DNA substrate, B, C = digestion products

To assess the gene editing in putative offtarget sites, genomic PCR amplification of the respective loci (Supplementary Table 3) followed by T7EI assay was performed. Both the CRISPR Design tool (Zhang lab, MIT) and the CRISPR Finder (Welcome Trust Sanger Institute) were used to identify potential offtargets.

### Restriction Enzyme Digestions

Restriction enzymes (all from NEB) were also used to assess gene editing. Digestion reactions for all restriction enzymes were incubated at 37 °C for 1 h in CutSmart Buffer with the exception of SspI that was incubated in NEB Buffer 2.1. Digestion products were visualised in 2% agarose gel in 1xTBE buffer.

### RNA extraction

Total RNA from cultured cells was prepared using the miRNeasy kit (Qiagen) as described previously^35^. In brief, cells were lysed with 700 μl of QIAzol reagent. Following a brief incubation at ambient temperature, 140 μl of chloroform were added and the solution was mixed vigorously. The samples were then centrifuged at 12,000 rpm for 15 min at 4 °C. The aqueous phase was carefully transferred to a new tube and 1.5 volumes of ethanol were added. The samples were applied directly to columns and washed according to the company’s protocol. Total RNA was eluted in 25 μl of nuclease free H_2_O

### MiRNA quantification

A total of 100 ng of RNA were reverse transcribed using the Megaplex Rodent Primer Pools A as previously described^27^. The reverse transcription (RT) reaction was performed according to the company’s recommendations (0.8 µl of Pooled Primers were combined with 0.2 µl of 100 mM dNTPs with dTTP, 0.8 µl of 10x Reverse-Transcription Buffer, 0.9 µl of MgCl_2_ (25 mM), 1.5 µl of Multiscribe Reverse-Transcriptase and 0.1 µl of RNAsin (20 U/µl) to a final volume of 7.5 µl). The RT reaction was set as follows: 16 °C for 2 min, 42 °C for 1 min and 50 °C for 1 sec for 40 cycles and then incubation at 85 °C for 5 min using a Veriti thermocycler (ThermoFisher Scientific, Runcorn, UK). The RT reaction products were diluted to 1 ng/µl corresponding RNA and stored at −20 °C. Taqman miRNA assays were used to assess the expression of mature miRNAs. For the quantitative polymerase chain reaction (QPCR) reaction, 2.25 µl of Megaplex reverse transcription product were combined with 0.25 µl of 20x Taqman miRNA Assay (ThermoFisher Scientific, Runcorn, UK) and 2.5 µl of the 2x Taqman Universal PCR Master Mix No Amp Erase UNG (ThermoFisher Scientific, Runcorn, UK) to a final volume of 5 µl. QPCR was performed on a Viia7 thermocycler at 95 °C for 10 min, followed by 40 cycles of 95 °C for 15 sec and 60 °C for 1 min. U6 was used as a normalization control.

### MiRNA Overexpression

Cells were plated at 60–70% confluency on the day before transfection. Mouse miRNA mimics and a nontargeting control were obtained by ThermoFisher Scientific, and transfected at a final concentration of 20 nM using Lipofectamine RMAiMAX (ThermoFisher Scientific, Runcorn, UK) as described previously^27^.

### Gene expression

QPCR was used to assess the gene expression levels. In these studies, 1 μg of RNA was reversed transcribed into cDNA using the High Capacity Reverse Transcriptase kit (ThermoFisher Scientific, Runcorn, UK). Prior to pri-miRNA assessment RNA samples were treated with DNAse for 30 min at 37 °C, to remove any genomic DNA contamination in the preparations. For all genes, Taqman Assays were used with the exception of Carmn that was assessed using specific primers^36^ (Supplementary Table 2) and SyBr Select Mastermix (ThermoFisher Scientific, Runcorn, UK). Beta Actin was used as a normalization control.

### Computational analysis

Secondary structures without pseudoknots were generated by RNAfold (Vienna RNA tools, http://rna.tbi.univie.ac.at/cgi-bin/RNAWebSuite/RNAfold.cgi) using the Turner model (Standard settings), while secondary structures with pseudoknots were generated by vsfold5^37^ (Chiba Institute of Technology, Japan, http://www.rna.it-chiba.ac.jp/~vsfold/vsfold5/) and Settings: 37 °C temperature; Kuhn length 6; Jacobson-Stockmayer gamma = 1.75 and contiguous stems = 6. Visualization of the secondary structures with pseudoknots was performed using the Pseudoviewer software (Inha University, S Korea http://wilab.inha.ac.kr/pseudoviewer/). The RNAComposer^38^ (Poland, http://rnacomposer.cs.put.poznan.pl/) was used to generate the pbd-files through molecular RNA simulation from vsfold5 output. The RNAComposer output pdb-files were then input to pyMOL to generate high quality 3D images. MiR-497a stem loop is depicted in green and its’ terminal loop in magenta. MiR-195a stem loop is depicted in red and its’ terminal loop in yellow.

### Statistical analysis

Statistical analyses were performed with the Student t-test with a Bonferroni post hoc test or ANOVA with Dunnett post hoc test, using GraphPad Prism 5 software. Results are shown as mean ± SD. A value of *P* < 0.05 was considered significant.

## Results

### Gene Editing of the MiR-497~195 Cluster

**Figure 1:**
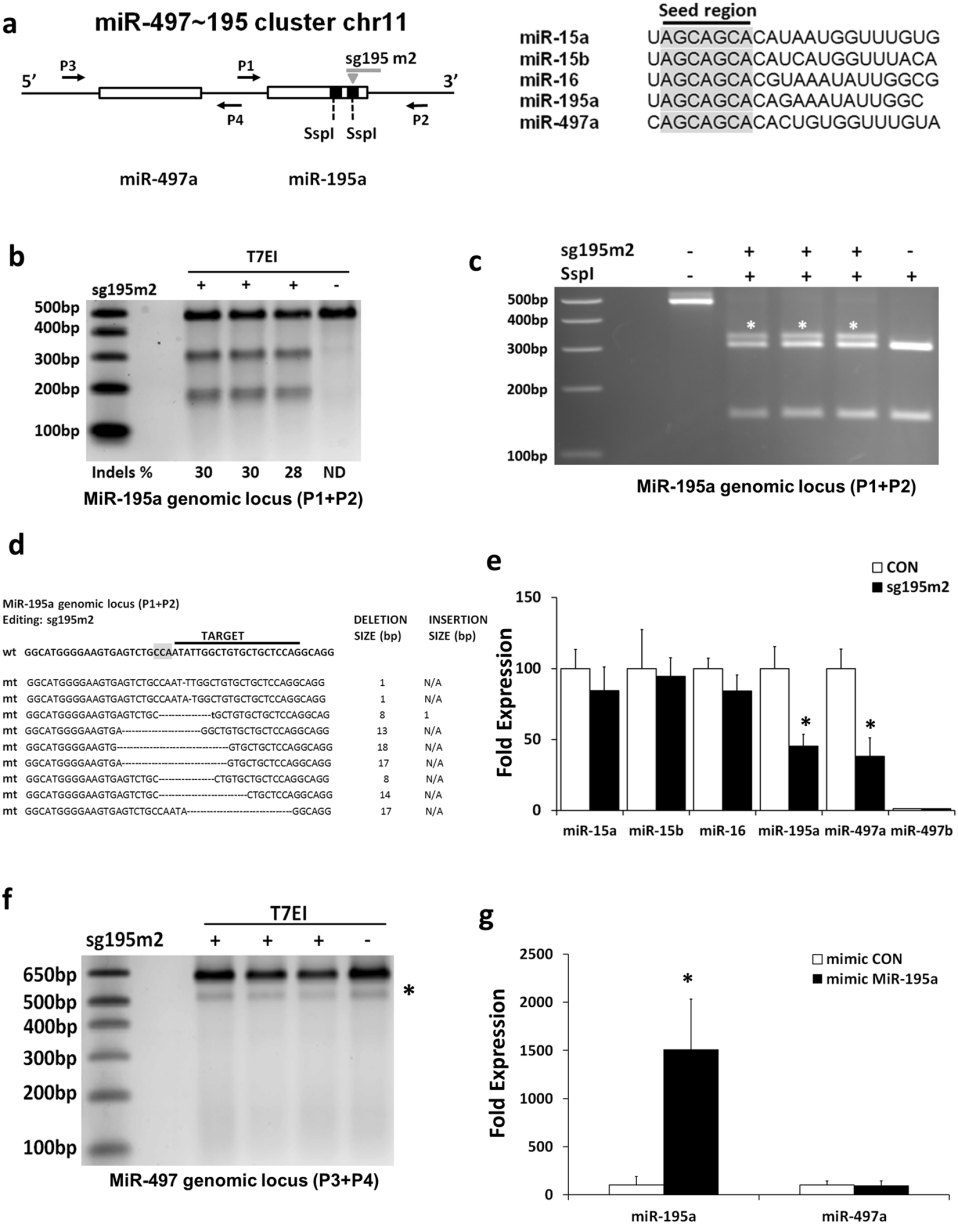
Gene editing of the miR-195a locus. (**a**) The genomic locus of the miR-497~195 cluster. MiR-195a forms a cluster with miR-497a and is transcribed as a single primary transcript. MiR-497b is encoded by the antisense strand and displays high homology with miR-497a but does not harbour the same seed sequence. Right panel, miR-15 family members share a common seed region (highlighted in grey) and display high sequence homology. (**b**) T7EI assay for the miR-195a locus. Three independent experiments were performed. ND: non detectable (**c**) The sg195m2 targets the miR-195a genomic locus and disrupts an SspI restriction site. Asterisks (*) indicate differential digestion products. (**d**) Sanger sequencing of the edited miR-195a genomic locus revealed the presence of random indels, mainly deletions, represented as dashes. The PAM sequence is highlighted in grey. Insertions are shown in bold small case fonts. N/A: Not Applicable. (**e**) Gene editing of the miR-195a locus resulted in the down-regulation of both miR-195a and miR-497a without affecting the expression of other members of the miR-15 family as assessed by qPCR. MiR-497b that is also encoded in the miR-497~195 cluster is not expressed in VSMCs. U6 was used as a normalization control. n = 3, *p < 0.05 Student’s t-test with Bonferroni post hoc test (**f**) T7EI assay for miR-497a genomic locus did not reveal any editing in the genomic locus of miR-497, *nonspecific T7EI digestion product. (**g**) QPCR quantification of mature miR-195a and miR-497a expression, following overexpression of miR-195a using miRNA mimics. U6 was used as a normalization control. n = 3, *p < 0.05 Student’s t test.

Cluster miR-497~195 is a homo-cluster composed of two members of the miR-15 family, miR-497a and miR-195a. The antisense strand encodes miR-497b, a miRNA that displays high homology with miR-497a but does not harbour the same seed sequence. In mice, the miR-15 family consists of miRNAs that display an identical seed region and high sequence homology overall^9, 27, 39^ (Fig. 1a, Supplementary Figure S1). MiR-195a has a prominent role in vascular remodelling and extracellular matrix deposition. Differential expression of miR-195 was also detected in the aneurysmal tissue while miR-195 manipulation was shown to regulate tissue remodelling^27^.

To determine whether targeting of the miR-195a hairpin could have an effect on the miR-497~195 cluster, we generated VSMCs stably expressing Cas9 nuclease. Guide RNA was designed using the online CRISPR Design Tool (http://tools.genome-engineering.org). The DNA sequence corresponding to the transcript annotated in miRBase v21 as stem loop miRNA was used as input sequence to design sgRNAs. The identified sg195m2 guide generates a DSB in the miR-195a stem loop that disrupts the SspI restriction digestion site (Fig. 1a, Supplementary Figure S1). Transfection of sg195m2 guide to VSMCs stably expressing Cas9 induced substantial gene editing (28–30%) as assessed by the T7 endonuclease I (T7EI) assay that identifies and cleaves mismatched DNA (Fig. 1b). As expected, editing of the locus led to disruption of the SspI restriction site and an altered digestion pattern (Fig. 1c). Mutation screening using Sanger sequencing demonstrated a panel of indels, mostly deletions, in the miR-195a locus (Fig. 1d). Using the RNAfold prediction tool, substantial alterations of the secondary structure of the miR-195a stem loop in mutants was identified (Supplementary Figure S1).

QPCR quantification indicated a 55% downregulation of the mature miR-195a expression in cells transfected with the sg195m2 guide (Fig. 1e), suggesting that editing of the locus led to a significant reduction in miR-195a expression. At the same time, no difference in miR-15a, miR-15b or miR-16 was observed indicating that the genomic editing did not lead to inhibition of the entire miR-15 family. No compensatory miRNA expression was observed either (Fig. 1e). MiR-497b was not expressed in VSMCs. Intriguingly, miRNA inhibition was not entirely specific, as a significant downregulation (62%) in miR-497a expression was observed (Fig. 1e). MiR-195a and miR-497a form a cluster on chromosome 11, are transcribed as one primary transcript, with both stem loops located within 500 bp. To determine whether the observed miR-497a downregulation was due to unexpected gene editing of the miR-497a genomic locus, we performed a T7EI assay. No specific T7 digestion products were detected suggesting that there is no editing in the locus (Fig. 1f). Further support to this notion was provided by Sanger sequencing as no mutations were detected (Supplementary Figure S2). Noteworthy, no editing in a panel of potential offtarget sites as predicted by the CRISPR Design tool (Zhang lab, MIT) and CRISPR Finder (Welcome Trust Sanger Institute) was detected (Supplementary Table 3, Supplementary Figure S3). Similar results were obtained using different guide (sg195 m3) to induce gene editing of the miR-195a stem loop. The significant downregulation of miR-195a was accompanied by a significant decrease in miR-497a expression levels (Supplementary Figure S4) with no editing in the miR-497 genomic locus (Supplementary Figure S5). No editing in potential offtarget sites for sg195 m3 was observed either (Supplementary Figure S5).

Downregulation of miR-497a may occur as a secondary effect due to the reduced levels of mature miR-195a. To determine whether the mature miR-195a expression was affecting the mature miR-497a or the miR-497a stem loop levels, gain and loss of function experiments can be performed. In miR-195a inhibition however, the sequence similarities between miR-195a and miR-497a indicate that the inhibitor can effectively silence both miRNAs (Supplementary Figure S6) and thus no conclusive data can be obtained. Overexpression of a miRNA can be achieved using short synthetic oligonucleotides that function as mature miRNA without requiring any processing by the Drosha or Dicer complexes. Following miR-195a overexpression using miRNA mimics, no differences on the expression of the mature miR-497a were observed (Fig. 1g), suggesting that the miR-497a expression is not controlled by miR-195a.

MiRNA biogenesis may depend on the primary miRNA structure and the accessibility of the stem loops to the enzymatic complexes of Drosha and Dicer^40^. Therefore we performed a computational analysis to determine whether structural constraints could impede the processing of the mutant primiRs. We focused on the analysis of the mutant 1 that harbours a deletion of 1 nucleotide (mut1), mutant 5 that harbours a deletion of 18 nucleotides (mut5) and the unedited transcript (wt). We used three software prediction programmes (RNAfold, Sfold, Co-fold) and all of them returned identical results. Secondary structures without pseudo-knots can be described as outerplanar graphs as they are 2D representations traversing the structure along backbone bonds that leave all hydrogen bonds at the same side of the direction (either all left-hand side or all right-hand side). Secondary structures with pseudo-knots expand significantly the search space for robust conformations and the above-mentioned property is no longer valid. Noteworthy, finding 2D presentations of structures with pseudo-knots by avoiding ‘crossing edges’ is much more complicated algorithmically than outerplanar representations. Interestingly, in our case the secondary structure predictions with pseudoknots indicated that wt and mut1 were similar but quite different from mut5 (Supplementary Figures S7 and S8).

**Figure 2:**
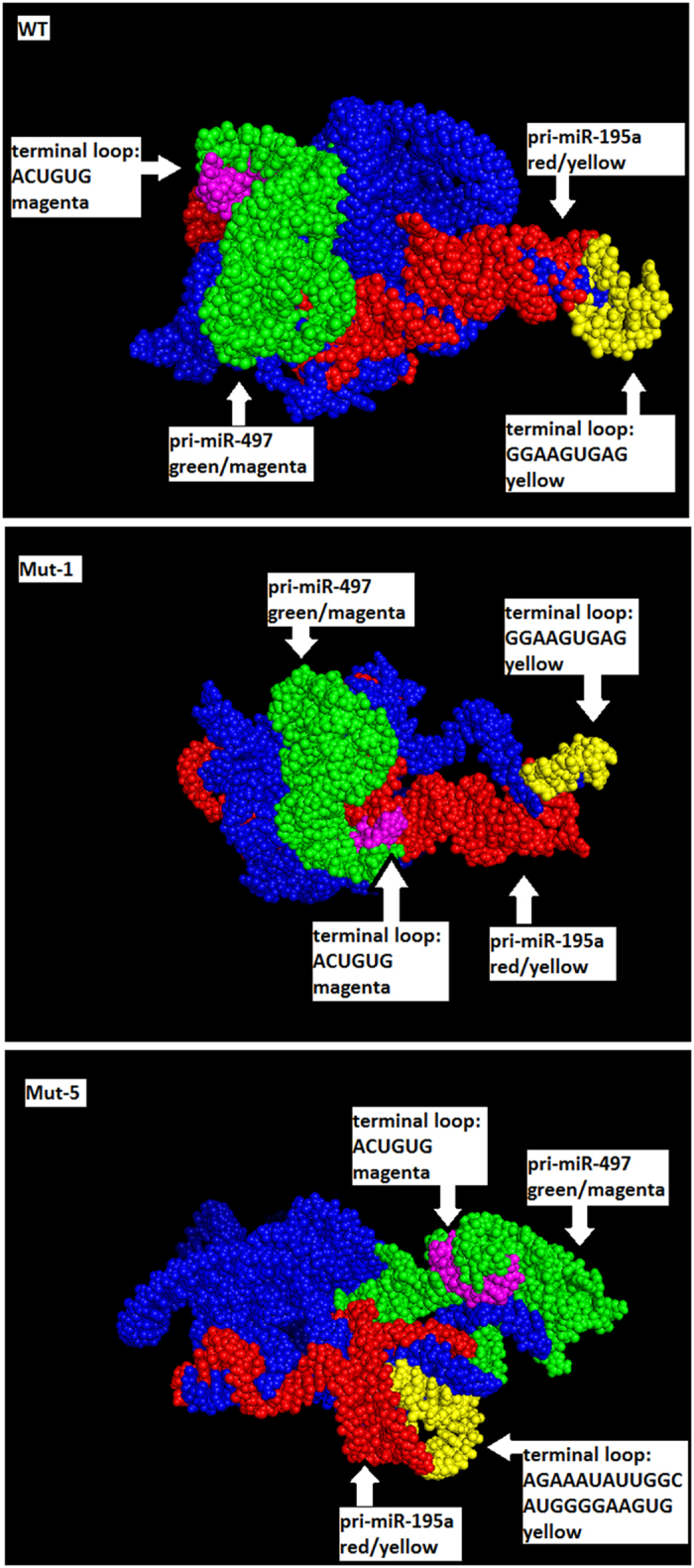
Effect of CRISPR/Cas9 editing in the tertiary structure of miR-497~195 cluster. Computational simulation of the 3D structure (generated by vsfold5 - with pseudoknots – as input to RNAComposer, pdb-file visualised by PyMOL) of the primiR-497~195 in unedited (wt), mutant harbouring a deletion of 1 nt (mut1) and a mutant harbouring a deletion of 18 nt (mut5). MiR-497a stem loop is depicted in green and its’ terminal loop in magenta. MiR-195a stem loop is depicted in red and its’ terminal loop in yellow.

The 3D simulation however highlighted clear differences. In the wt, the miR-195a stem loop was prominent while the miR-497a stem loop more compressed but clearly accessible. In the mut1, the conformation of miR-195a stem loop was similar although the single nucleotide mutation had an effect leading to a miR-497a stem loop now strongly attached to the main core and not as accessible as in wt. Nevertheless, profound differences were observed in the conformation of mut5. No typical hairpin for primiR-195a could be detected and the structure was more strongly entangled with the main core. The miR-497a stem loop displayed a compact shape in close proximity with the miR-195a stem loop but relatively distal from the main core and with no clear accessibility to the hairpin (Fig. 2). A movie reconstruction of the tertiary structure of the wt and mutant primiR-497~195 is provided in Supplementary Figure S9. Overall, the 3D remodelling indicates a clear difference between the entire structure of wt and mut5 and to a lesser extent also between wt and mut1 with accessibility of the miR-497a stem loop being affected in both mutants. These results suggest that extensive deletions (in this case 18 nt) in the genomics locus of miR-195 stem loop can alter the tertiary structure of the entire miR-497~195 transcript.

### Gene Editing of the MiR-143~145 Cluster

**Figure 3:**
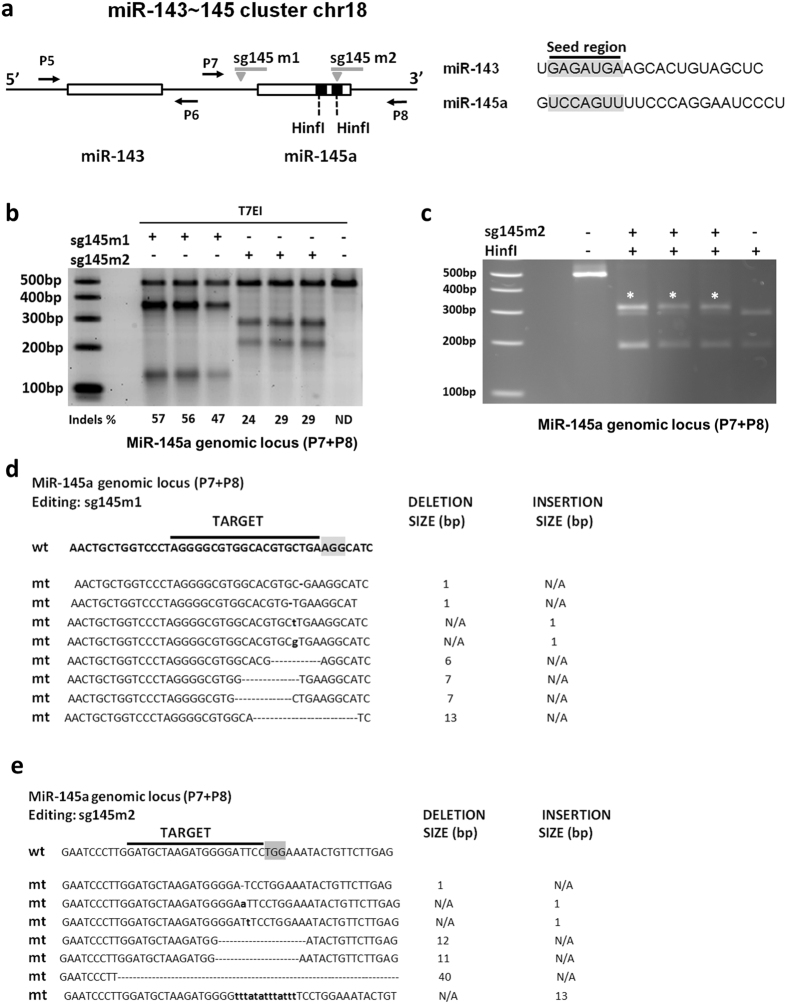
Gene editing of the miR-145a locus. (**a**) The genomic locus of the miR-143~145 cluster. MiR-145a forms a cluster with miR-143 and is transcribed as a single primary transcript. Right panel, miR-145 and miR-143 do not share a common seed region (highlighted in grey) and display no sequence homology. (**b**) T7EI assay for the miR-145a locus. Three independent experiments were performed. ND: non detectable (**c**) The sg145m2 targets the miR-145 genomic locus and disrupts an SspI restriction site. Asterisks (*) indicate differential digestion products. (**d**,**e**) Sanger sequencing of the edited miR-145a genomic locus using sg145m1 and sg145m2, respectively. The PAM sequence is highlighted in grey. Deletions are represented as dashes, insertions are shown in bold small case fonts. N/A: Not Applicable.

To determine the effect of gene editing in a hetero-cluster we focused on the miR-143~145 cluster^41^. This locus that spans ~1400 bp in mice harbours miR-143 and miR-145a, two miRNAs that do not share sequence similarities (Fig. 3a, Supplementary Figure S10) but regulate essential functions for VSMCs growth, differentiation, and contractility^42, 43^. Two sgRNAs targeting the miR-145a locus were designed. Sg145m2 guide targets the miR-145a stem loop and disrupts a HinfI restriction site, while sg145m1 guide generates a DSB in close proximity, 30 bp upstream of the miR-145a stem loop (Fig. 3a, Supplementary Figure S10). T7EI results indicate that the sgRNA145m1 guide was more efficient in inducing gene editing with indels ranging from 47–57%, while sgRNA145m2 displayed editing efficiency of 24–29% (Fig. 3b). Digestion with HinfI confirmed the editing following sg145m2 guide introduction (Fig. 3c). Sanger sequencing demonstrated small indel formation (1–40 bp) with either sgRNA (Fig. 3d,e).

**Figure 4:**
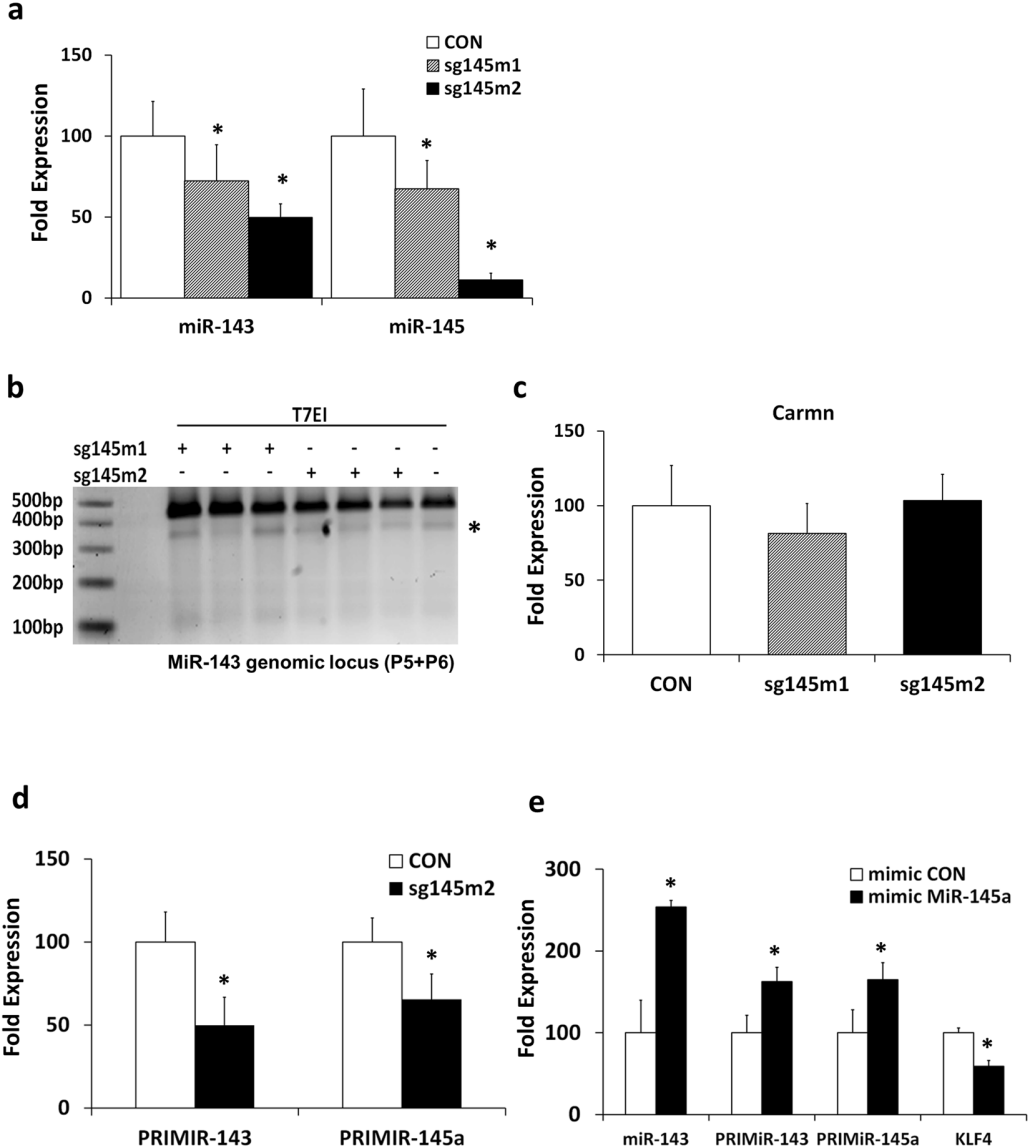
Effect of CRISPR/Cas9 editing of the miR-143~145 cluster on miRNA expression. (**a**) QPCR quantification of mature miRNA expression following gene editing of the miR-145a locus. U6 was used as a normalization control. n = 3, *p < 0.05 (ANOVA with Dunnett post hoc test) (**b**) T7EI assay for the miR-143 genomic locus. *nonspecific T7EI digestion product. (**c**) QPCR quantification of Carmn in sg145m2 edited cells. Beta actin was used as a normalization control. (n = 3) (ANOVA with Dunnett post hoc test) (**d**) QPCR quantification of the primiR-143~145 expression in sg145m2 edited cells. Pri-miRNA Taqman assays that are specifically designed to amplify flanking sequences within 500 base pairs on either side of the stem loop were used. Beta actin was used as a normalization control. n = 3, *p < 0.05. (**e**) The expression of the mature miR-143 and primiR-143~145, following miR-145a overexpression with miR-145a mimics, was quantified by qPCR. Kruppel Like Factor 4 (KLF4), a validated target of miR-145a was also assessed. Beta actin was used as a normalization control. n = 3, *p < 0.05 (ANOVA with Dunnett post hoc test).

Gene editing had a robust effect on miRNA expression. A strong inhibition of mature miR-145a and miR-143 (89% and 50%, respectively) expression was observed following sg145m2 application (Fig. 4a). Interestingly, despite the high levels of gene editing induced by the sgRNAm1 guide the downregulation in mature miR-145a and miR-143 expression was significant but lower than sgRNAm2 (33% and 28%, respectively), suggesting that targeting directly the stem loop is a more efficient strategy for inhibiting miRNA expression (Fig. 4a). Further support to this notion was provided by Sanger sequencing. The sg145m1 guide led to indels that did not disrupt the miR-145a stem loop sequence, while sg145m2 guide led to mutations in the miR-145a stem loop that also alter its secondary structure (Supplementary Figure S10). Despite the significant decrease in miR-143 expression, no editing of the miR-143 genomic locus was observed with either guide (Fig. 4b). Mutation screening revealed no indels in the miR-143 locus in edited cells with either guide (Supplementary Figure S11). Intriguingly, the expression of Carmn (cardiac mesoderm enhancer-associated non-coding RNA), a long non coding RNA overlapping the miR-143~145 cluster (Supplementary Figure S12, Fig. 4c) that constitutes an independent transcription unit^36^ did not differ in miR-145 edited cells confirming that only the primary transcript (primiR-143~145) is affected. In regards to the tertiary structure of the cluster miR-143~145, as it is length is about 1400 bp, it cannot be analysed using the algorithms currently available for the computational simulation. The development of more elegant tools to predict the tertiary structure of longer sequences will provide further insights.

To elaborate on the regulatory mechanisms involved, we quantified the expression of primiR-143 and primiR-145 in sg145m2 edited cells. To this end, we used commercially available assays that are designed to target the flanking sequences on either side of the miRNA stem loop. In essence, the primiR-143 and primiR-145a assays that we used targeted the hairpin of miR-143 and miR-145a, respectively. A sharp decrease was observed indicating that targeting miR-145a affects the expression of the entire primiR-143~145 (Fig. 4d).

In order to determine whether mature miR-145a expression exerts transcriptional control on the miR-143~145 cluster, we overexpressed miR-145a in VSMCs using miRNA mimics. These are short synthetic oligonucleotides that function as mature miRNA without requiring any processing by the Drosha or Dicer complexes. High levels of miR-145a led to increased miR-143 expression (250%) that was accompanied by a coordinated increase in primiR-143 and miR-145a expression (162% and 165% respectively), indicating transcriptional regulation. As expected the levels of KLF4, a validated target of miR-145a^41^ were significantly downregulated (40%) following miR-145a overexpression (Fig. 4e). These data suggest that the CRISPR/Cas9 editing technology is extremely precise, indels occurred only in the vicinity of the DSB as no mutations were identified in a 1400 bp region that encodes the cluster. The use of CRISPR/Cas9 platform for miRNA inhibition can reveal novel regulatory mechanisms for clustered miRNA expression.

### Gene Editing of the MiR-17~92 and MiR-106~25 Clusters

**Figure 5:**
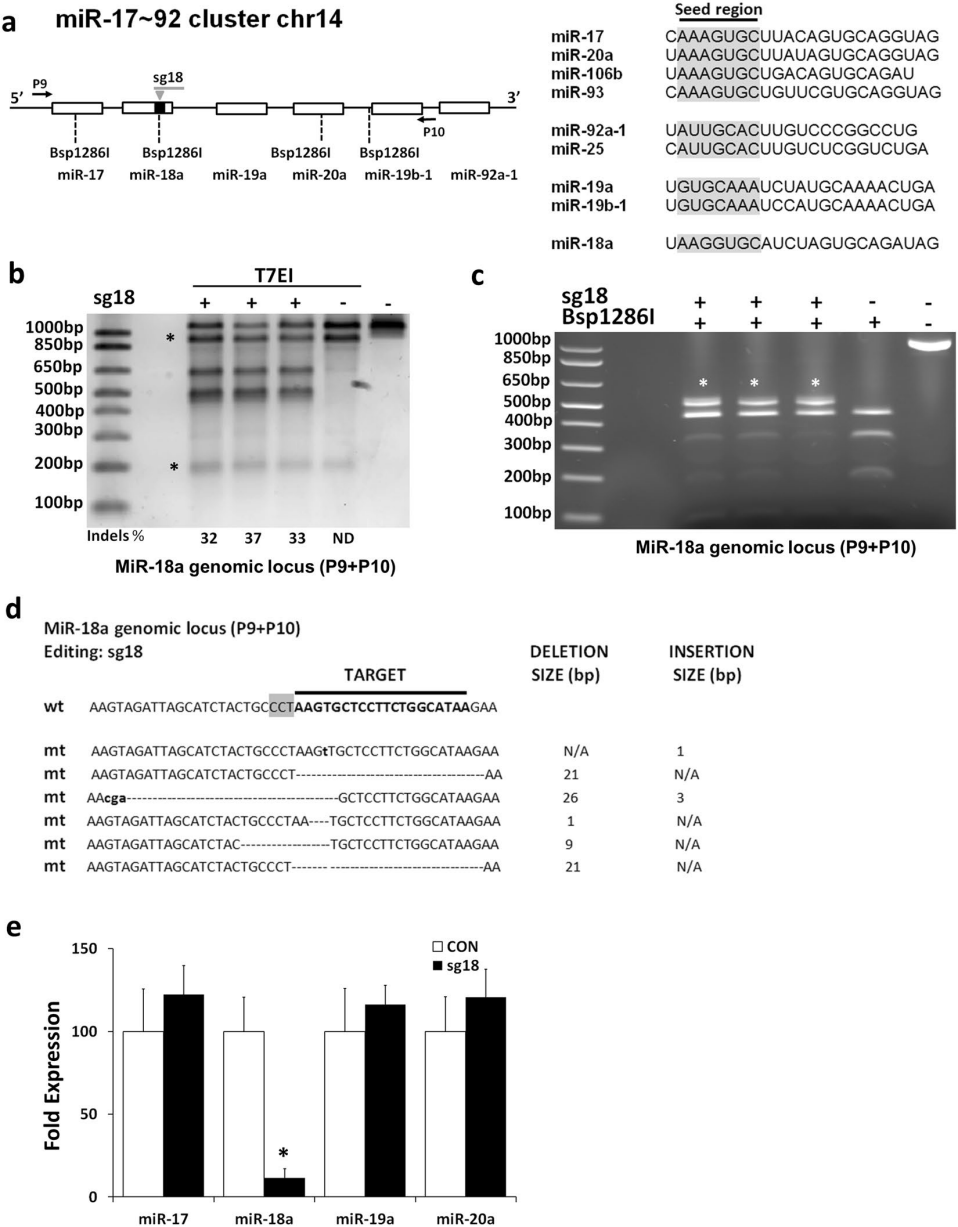
Gene editing of the miR-18a locus. (**a**) The genomic locus of the miR-17~92 cluster. Right panels, miRNAs in this cluster are derived from four different families that share different degrees of homology (seed region highlighted in grey). (**b**) T7EI assay for the miR-17~92 cluster. Three independent experiments are shown. ND: non detectable. Asterisks (*) indicate nonspecific T7EI digestion products. (**c**) The sg18 targets the miR-18a genomic locus and disrupts a Bsp1286I restriction site. Asterisks (*) indicate differential digestion products. (**d**) Sanger sequencing of the edited miR-18a genomic locus. The PAM sequence is highlighted in grey. Deletions are represented as dashes. Insertions are shown in bold small case fonts. N/A: Not Applicable. (**e**) QPCR quantification of mature miRNA expression following editing with sg18. U6 was used as a normalization control. n = 3, *p < 0.05 (Student’s t-test with Bonferroni post hoc test).

We next focused on two highly studied miRNA clusters in the mouse genome, namely miR-17~92 and miR-106b~25 spanning 800 bp and 300 bp respectively. The miR-17~92 cluster plays a pivotal role in the cardiovascular system and in cancer and mediates processes such as angiogenesis^10, 44^. This cluster consists of six miRNAs that can be grouped in four families based on their seed sequence (Fig. 5a, Supplementary Figure S13). Intriguingly, two paralogs, the miR-106a~363 and miR-106b~25 clusters believed to be derived from a series of duplication and deletion events during vertebrate evolution have been identified^45^. Gene editing using sg18 that targets the miR-18a stem loop (Figure 5a, Supplementary Figure S13) was efficient (32–37%) as assessed by T7EI assay (Fig. 5b) and disrupted the Bsp1286I restriction site (Fig. 5c). Sanger sequencing revealed the presence of small deletions in the miR-18a stem loop (Fig. 5d) that were predicted to induce a differential secondary conformation (Supplementary Figure S13). As previously, these deletions were very specific and occurred only in the vicinity of the DSB. No mutations were identified in a 1 kb region that encodes the cluster (Supplementary Figure S14). In the case of miR-17~92 cluster, the expression of miR-18a diminished robustly (88%) but there was no impact on miR-17, miR-19a and miR-20a levels, all miRNAs located in the cluster (Fig. 5e). The expression of miR-19b and miR-92a was not assessed as they are also encoded by a second miRNA cluster on chromosome X (Supplementary Table 1).

**Figure 6:**
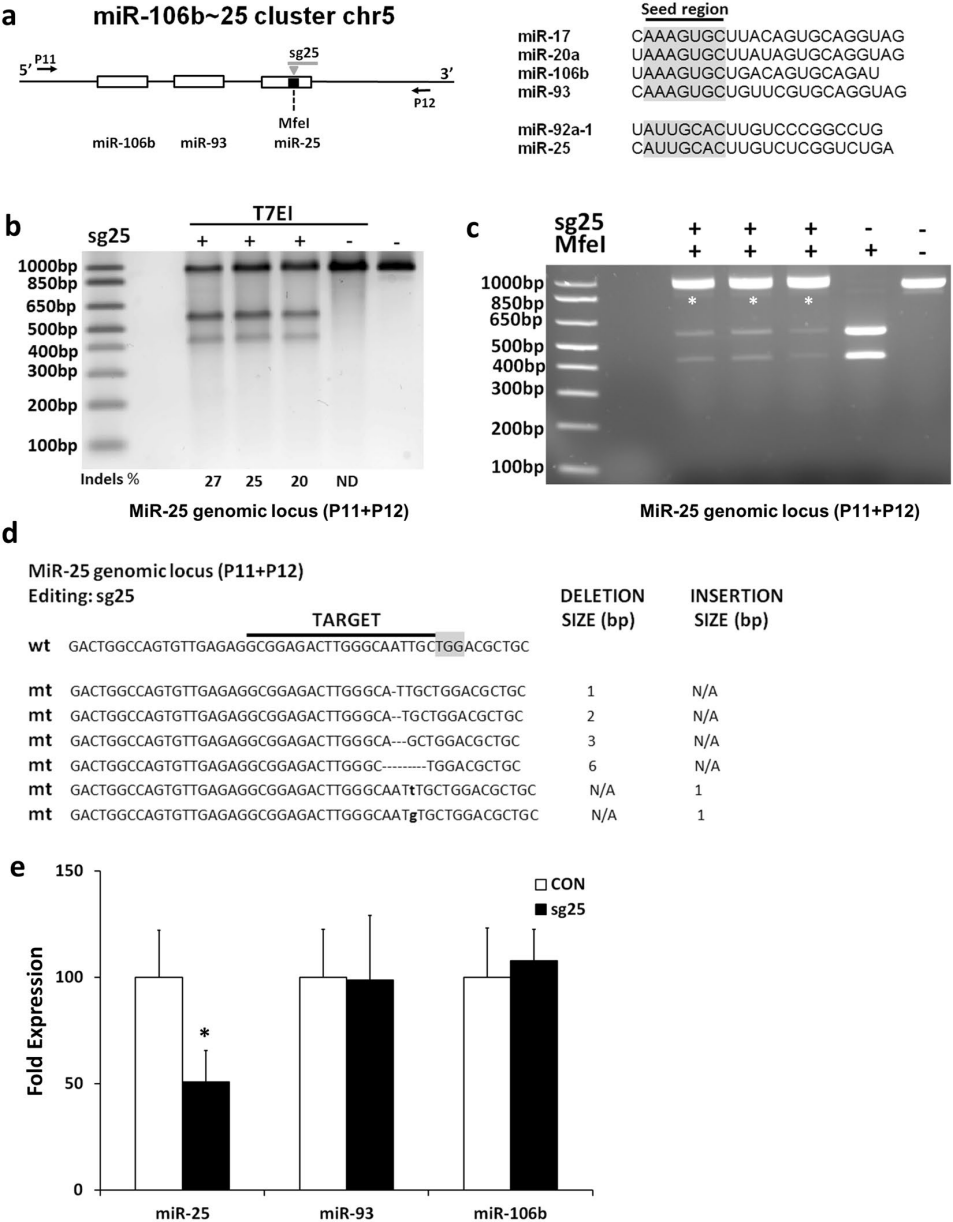
Gene editing of the miR-25 locus. (**a**) The genomic locus of the miR-106b~25 cluster. Right panel, miRNAs in this cluster are derived from two different families that share varying degrees of homology (seed region highlighted in grey). (**b**) T7EI assay for the miR-106b~25 cluster. ND: non detectable. (**c**) The sg25 targets the miR-25 genomic locus and disrupts a MfeI restriction site. Asterisks (*) indicate undigested amplicons. (**d**) Sanger sequencing of the edited miR-25 genomic locus. The PAM sequence is highlighted in grey. Deletions are represented as dashes, insertions are shown in bold small case fonts. N/A: Not Applicable. (**e**) QPCR quantification of mature miRNAs following editing with sg25. U6 was used as a normalization control. n = 6, *p < 0.05 (Student’s t-test with Bonferroni post hoc test).

Similar results were obtained when the stem loop of miR-25 was targeted in the miR-106b~25 cluster (Fig. 6a, Supplementary Figure S15). Effective editing was observed with sg25 (20–27%) as determined by T7EI assay (Fig. 6b) or the disruption of the MfeI restriction digestion site (Fig. 6c, Supplementary Figure S15). Sanger sequencing demonstrated a mutation pattern of small indels (Fig. 6d) that led to alteration in the secondary structure of the miR-25 stem loop (Supplementary Figure S15). No editing was observed for the miR-93 and miR-106b loci (Supplementary Figure S16). QPCR quantification demonstrated a significant downregulation of miR-25 expression (49%) in edited cells (Fig. 6e) while the levels of miR-93 and miR-106b were not affected.

## Discussion

MiRNAs provide rapid responses to counteract rapid and variable fluctuations and safeguard the robustness of biological systems^46^. A large portion of miRNAs organise in miRNA clusters^5^. Computational analysis suggests that the existence of miRNA clusters is much higher than expected by chance^5^. Based on their regulation, they can be divided to homo-clusters, that are composed of miRNA members of the same family and control their targets in a single step thus resulting in a rapid regulation and hetero-clusters that are composed of miRNA members of different families that typically exert control of their targets in multiple steps in a more delayed response^6^. MiRNA clusters often undergo a series of duplication events that lead to the generation of paralogues that are thought to regulate a similar set of genes and have overlapping functions^6^.

Here we interrogated the use of gene editing as a tool to understand the clustered miRNA regulation. Our study focused on four clusters, the homo-cluster miR-497~195, the hetero-clusters miR-143~145 and miR-17~92 and cluster miR-106b~25 a paralogue of cluster miR-17~92. The CRISPR/Cas9 system emerged as a very precise tool, inducing indel formation only in the vicinity of DSB. Our data demonstrate a diverse panel of regulatory mechanisms of expression for clustered miRNAs. Intriguingly, we found that in miR-497~195 cluster mutations in miR-195a hairpin can affect the expression of miR-497a that resides in the other hairpin of the same cluster. Computational analysis highlighted differences in the tertiary structure of the primiRNA for mutant with extensive deletions that may contribute to the differential mature miRNA expression. Overall, we found that in spite of the sequence diversity of primiRNAs, mutations are well tolerated provided that they don’t disrupt critical elements such as stem length, bulge position and terminal loops. Furthermore, we demonstrate that although miRNAs in a cluster form co-transcriptional unit their expression is not always interdependent.

MiRNA maturation is a complex process. In the canonical pathway the first step is a cleavage event in the nucleus by RNAse III DROSHA and its co-factor DGCR8 forming the microprocessor, a bulky complex of 364 kDa^47^. A very specific and precise recognition of the primiRNA by the microprocessor emerged in a model whereby a trimeric complex is formed encompassing one DROSHA that serves as a ruler by recognizing basal elements and two DGCR8 adaptors that interact with the apical elements to ensure fidelity of processing^47^. This modular model predicts varying contributions of several determinants that need to interact in a coordinated response for primiRNA recognition^48^.

Elegant experimental approaches have identified additional recognition and processing features^49, 50^. Secondary structures such as stem length, hairpin pairing, bulge size and position and apical loop size were shown to contribute to effective miRNA biogenesis. Moreover, sequence motifs, such as a UG motif at the base of the hairpin and a UGU/GUG motif in the apical loop and a CNNC motif downstream of the hairpin, can enhance processing^48, 49, 51^. Importantly, these primary sequence motifs exert their effects in some primiRNAs but not in others and are thought to have an additive effect in primiRNA processing.

An intricate set of rules and modifications that are preferentially utilized in miRNAs over non-miRNA hairpins were also uncovered^50^. An optimal stem length and two bulge depleted regions in the miRNA hairpin stem that may function as protein-interacting surfaces were proposed. The presence of bulges in the depleted sites was dimmed as detrimental. Moreover, the CNNC motif was found to be important for optimal length hairpins. These findings led to the prediction and experimental validation for a significant role of single-nucleotide polymorphisms (SNPs) in altering primiRNA processing and miRNA biogenesis in several occasions^50^. Adding to the complexity of miRNA biogenesis, RNA binding proteins (RBPs) can post-transcriptionally modify various steps and affect the miRNA processing^52^. Employing a large scale biochemical screen, a stimulatory or inhibiting role for RBPs in miRNA processing was recently revealed^26^.

These findings are intriguing and imply that CRISPR/Cas9 gene editing of miRNA genes can affect processing of the hairpin in a dual manner. Directly through sequence alterations and disruption of sequence motifs but also structurally, through changes in the stem length, bulge size and position and terminal loop length. In the case of clustered miRNAs, our data imply that the tertiary structure may also contribute to the processing of the primiRNA to individual mature miRNAs. Computational simulation demonstrated that disruption of the miR-195a stem loop may have imposed tertiary constraints that impede effective processing of the entire primary transcript leading to diminished mature miR-497a levels. In a bioinformatic reconstruction of a mutant miR-195a stem loop harbouring a 18nt deletion a clear difference was observed compared to the wild type. Noteworthy, a UGU/GUG motif in the apical loop is not present in the miR-195a stem loop. Nevertheless, a direct disruption of terminal loop both in terms of sequence and in length was detected in the secondary structure of the mutant. Our findings are in line with previous reports that demonstrated that mutations in the terminal loop abrogate efficient primiRNA processing^53^. Further support to this notion is provided by studies that show an autoregulatory role for the tertiary structure of primiR-17~92 in its maturation^40, 54^ and binding of auxiliary factors to conserved terminal loops.

Mutation of primiRNA sequences are well tolerated provided that they do not disrupt critical elements. In the case of miR-143~145, introducing loxP sites for Cre-mediated recombination in the miR-143 or miR-145 genomic loci resulted in miR-143 and miR-145a mutant mice that do not exhibit an effect on the expression of the miR-145a and miR-143 respectively^43^. On the other hand, replacing the miR-143 genomic region with a LacZ reporter disrupted miR-143 expression but also led to effective silencing of the miR-145a expression^55^. It is clear that the two approaches differ substantially. While both disrupt the miRNA gene, the introduction of a reporter in the clustered primary transcript renders it unsuitable for downstream processing and the maturation cascade for miR-145a generation, despite the presence of an intact miR-145a stem loop. It is tempting to speculate that in this case as well the tertiary structure functions as the critical determinant of mature clustered miRNA biogenesis. Nevertheless, it is clear that the introduction of mutations in the primiR-143~145 per se does not hamper miRNA maturation^43^. This is in line with our finding that sg145m1 has a minimal effect on miR-143 and miR-145a expression despite inducing effective editing of the locus.

Our findings imply a co-ordination in the maturation of clustered miRNAs. In the case of the miR-143~145 cluster we also detected interdependency in the mature miRNA expression. Gene editing of miR-145a led to decreased expression of miR-145a and miR-143 and miR-145a mimics resulted in increased levels of primiR-143 and mature miR-143 indicating transcriptional regulation and a feed-forward loop that reinforces the expression of the entire cluster. We provide direct evidence for the role of mature miR-145a in triggering the expression of the cluster miR-143~miR-145. Although it is difficult to dissect whether the differences in miR-143 expression in our edited cells are due to structural changes that hamper the processing of the cluster primiRNA or due to transcriptional regulation triggered by the reduced miR-145a levels, the two possibilities are not mutually exclusive. It is important to point out that miR-143 and miR-145a show no sequence homology suggesting that they do not have common targets^41^. Nonetheless, both miRNAs act as part of a network that controls cytoskeletal remodelling and phenotypic switching of VSMCs under pathological conditions^43^. This underlines the added value of miRNA clusters that provide an effective mechanism of cellular response and may explain the evolutionary pressure for their sequence conservation.

Interestingly, interdependency in expression does not seem to be a common feature in all clustered miRNAs. Our gene editing in cluster miR-17~92 and miR-106b~25 revealed that targeting of miR-18a and miR-25 does not affect the expression of other miRNAs in each cluster. No compensatory increase of these miRNAs was observed in edited cells either. Overexpression of miR-195a also did not result in enhanced levels of miR-497a suggesting no interdependency in the miR-497~195 cluster. Thus it seems that while coordinated expression of miRNAs in clusters is a shared feature, feed-forward and feedback loops are in place only when a synergistic effect and combined regulation of multiple pathways is required.

In conclusion, CRISPR/Cas9 emerged as a powerful tool to dissect the regulation and function of clustered miRNAs. The technology provides a unique platform to establish mutant cell lines or mouse models and elucidate the specific contribution of miRNA families in cellular responses both at baseline and following injury.

## Electronic supplementary material

Supplementary Info
