# Supplementary material for: CRISPR/Cas9 editing reveals novel mechanisms of clustered microRNA regulation and function
Source: Sci Rep. 2017 Aug 17;7:8585. doi: 10.1038/s41598-017-09268-0 (PMC5561095; doi:10.1038/s41598-017-09268-0)

ONLINE SUPPLEMENT

CRISPR/Cas9 editing reveals novel mechanisms of clustered microRNA regulation and function

Lazaros Lataniotis[1], Andreas Albrecht[2], Fatma O. Kok[3], Clinton A.L. Monfries[4], Lorena Benedetti[5], Nathan D. Lawson[3], Simon M. Hughes[4], Kathleen Steinhofel[6], Manuel Mayr[1] and Anna Zampetaki[1]

1 King’s British Heart Foundation Centre, King’s College London, UK.
2 Middlesex University, School of Science and Technology, London, UK.
3 University of Massachusetts Medical School, Department of Molecular, Cell, and Cancer Biology, Worcester, USA.
4 King's College London, Randall Division, London, UK.
5 King's College London, Division of Cancer Studies, London, UK.
6 King's College London, Informatics Department, London, UK.

Supplementary Figure S1

A

```
CLUSTAL W (1.83) multiple sequence alignment

miR-15a        UAGCAGCACAUAAUGGUUUGUG
miR-15b        UAGCAGCACAUCAUGGUUUACA
miR-16-1       UAGCAGCACGUAAAUAUUGGCG
miR-16-2       UAGCAGCACGUAAAUAUUGGCG
miR-195a       UAGCAGCACAGAAAUAUU-GGC
miR-497a       CAGCAGCACACUGUGGUUUGUA
                ********        **

MiR-497a       CAGCAGCACACUGUGGUUUGU-----A
MiR-497b       ---CACCACAGUGUGGUUUGGACGUGG
                  ** **** *********
```

B

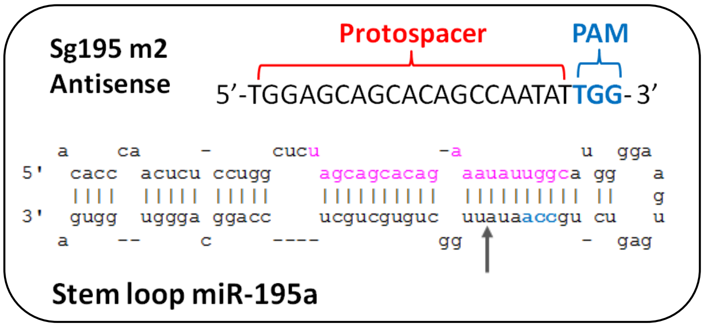

C

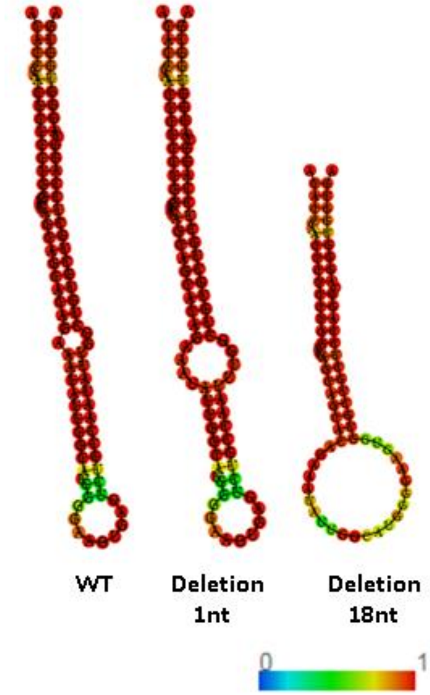

Supplementary Figure S1. A. Sequence Alignment. The T-Coffee multiple sequence alignment program was used to compare the mouse miR-15 family members. Right Panel: miR-497a displays high homology with miR-497b. MiR-497b is encoded by the antisense strand of the miR-497a~195 cluster and does not harbour the same seed sequence. **B. Schematic representation of sg195 m2.** The DNA sequence corresponding to the transcript annotated in miRBase v21 as stem loop miRNA was used as input sequence to the CRISPR Design tool. PAM sequence is highlighted in blue. The mature miRNA sequence is highlighted in purple and arrow indicated the site of mutations induced by random indels following editing of the genomic locus in mutant cells. **C. Minimum free energy structure of the miR-195a stem loop loop following sg195m2 editing as assessed by the structure prediction software RNAfold.** The base-pairing probability, as indication of structural remodelling or dynamics, was used for color-coding.

Supplementary Figure S2

MiR-497a genomic locus (P3+P4)
Editing: sg195m2

```
wt   CCTGCCCCGCCCCAGCAGCACACTGTGGTTTGTACGGCACTGTGGCCACGTCCAAACCACACTGTGGTGTTAGAGCGAGGGTA
1    CCTGCCCCGCCCCAGCAGCACACTGTGGTTTGTACGGCACTGTGGCCACGTCCAAACCACACTGTGGTGTTAGAGCGAGGGTA
2    CCTGCCCCGCCCCAGCAGCACACTGTGGTTTGTACGGCACTGTGGCCACGTCCAAACCACACTGTGGTGTTAGAGCGAGGGTA
3    CCTGCCCCGCCCCAGCAGCACACTGTGGTTTGTACGGCACTGTGGCCACGTCCAAACCACACTGTGGTGTTAGAGCGAGGGTA
4    CCTGCCCCGCCCCAGCAGCACACTGTGGTTTGTACGGCACTGTGGCCACGTCCAAACCACACTGTGGTGTTAGAGCGAGGGTA
5    CCTGCCCCGCCCCAGCAGCACACTGTGGTTTGTACGGCACTGTGGCCACGTCCAAACCACACTGTGGTGTTAGAGCGAGGGTA
6    CCTGCCCCGCCCCAGCAGCACACTGTGGTTTGTACGGCACTGTGGCCACGTCCAAACCACACTGTGGTGTTAGAGCGAGGGTA
7    CCTGCCCCGCCCCAGCAGCACACTGTGGTTTGTACGGCACTGTGGCCACGTCCAAACCACACTGTGGTGTTAGAGCGAGGGTA
8    CCTGCCCCGCCCCAGCAGCACACTGTGGTTTGTACGGCACTGTGGCCACGTCCAAACCACACTGTGGTGTTAGAGCGAGGGTA
9    CCTGCCCCGCCCCAGCAGCACACTGTGGTTTGTACGGCACTGTGGCCACGTCCAAACCACACTGTGGTGTTAGAGCGAGGGTA
10   CCTGCCCCGCCCCAGCAGCACACTGTGGTTTGTACGGCACTGTGGCCACGTCCAAACCACACTGTGGTGTTAGAGCGAGGGTA
     ***********************************************************************************
```

Supplementary Figure S2. Clustal alignment of Sanger sequencing of the miR-497a stem loop locus following sg195m2 editing in VSMCs. Wt: unedited cells, 1-10 randomly picked individual colonies harboring genomic PCR amplicons from sg195m2 edited VSMCs. P3, P4: Genomic PCR primers.

Supplementary Figure S3

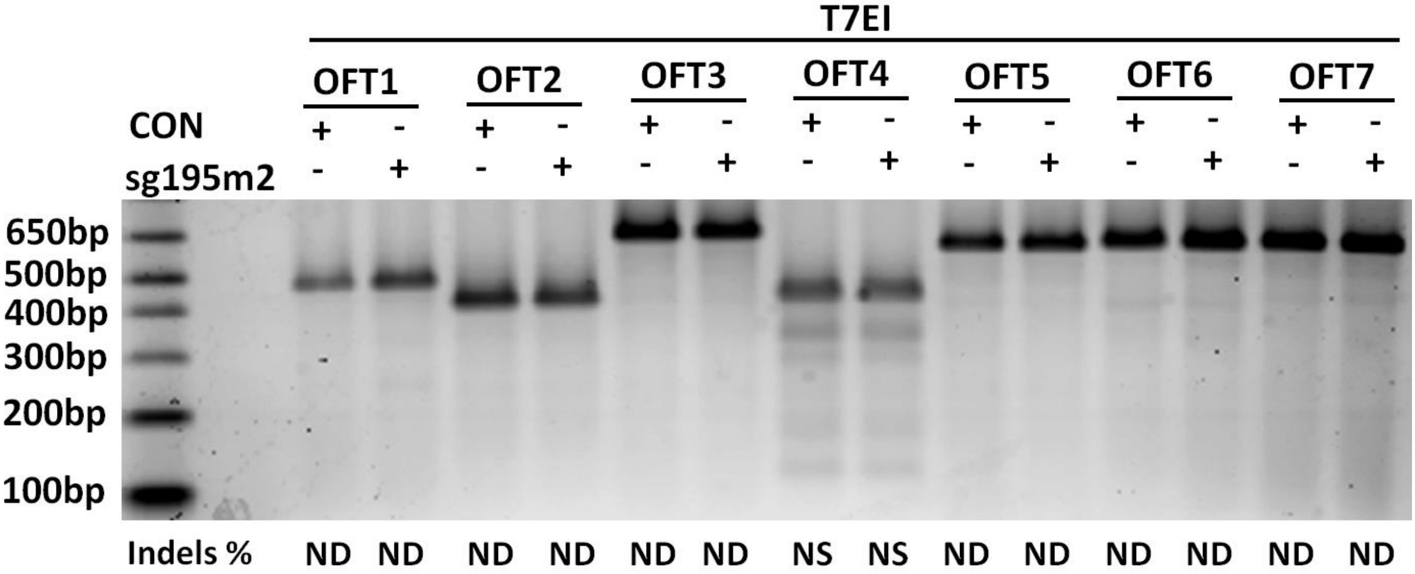

Supplementary Figure S3. Sg195 m2 OFFTARGETS. T7EI assessment of gene editing in the loci of putative OFFTARGET sites for sg195 m2 as predicted by the CRISPR design tool (Zhang Lab, MIT) and CRISPR Finder (Welcome Trust Sanger Institute). OFT; OFFTARGET, ND: Non Detectable, NS: Non Specific.

Supplementary Figure S4

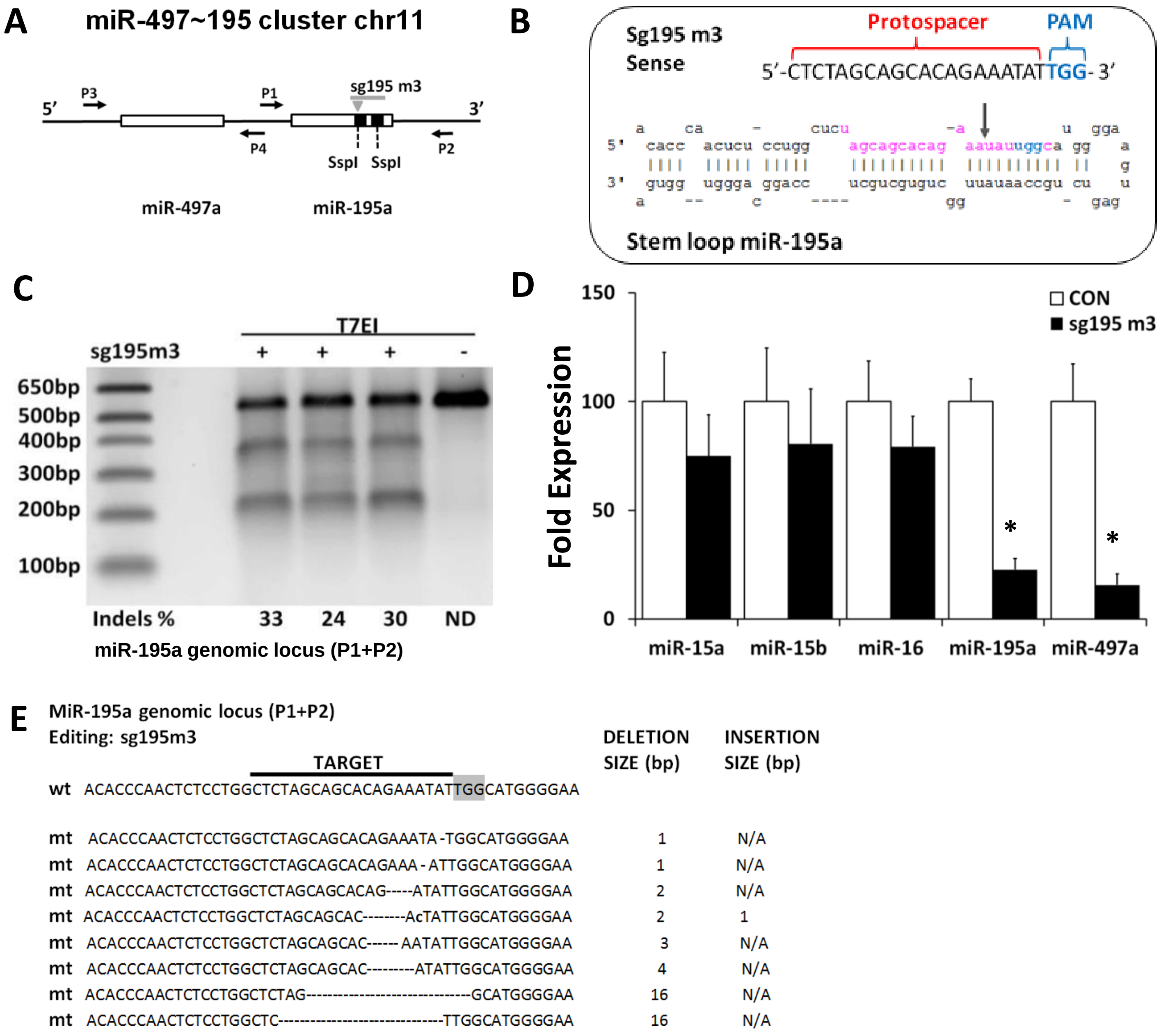

Supplementary Figure S4. Gene editing of the miR-195a locus using sgRNA195 m3. A. The genomic locus of the miR-497~195 cluster. B. Schematic representation of sg195 m3. The DNA sequence corresponding to the transcript annotated in miRBase v21 as stem loop miRNA was used as input sequence to the CRISPR Design tool. PAM sequence is highlighted in blue. The mature miRNA sequence is highlighted in purple and arrow indicated the site of mutations induced by random indels following editing of the genomic locus in mutant cells. **C. T7EI assay for the miR-195a locus following editing with sg195m3.** ND: Non detectable. P1, P2: Genomic PCR primers. **D. Gene editing of the miR-195a locus resulted in the down-regulation of both miR-195a and miR-497a without affecting the expression of other members of the miR-15 family as assessed by qPCR.** U6 was used as a normalization control. n=4, * $p<0.05$. **E. Sanger sequencing of the sg195 m3 edited miR-195a genomic locus.** The presence of random indels, mainly deletions was detected and represented as dashes. The PAM sequence is highlighted in grey. Insertions are shown in bold small case fonts. N/A: Not Applicable.

Supplementary Figure S5

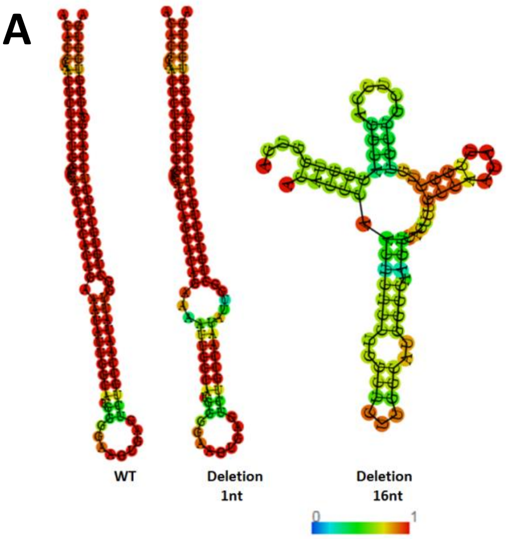

B MiR-497a genomic locus (P3+P4)
Editing: sg195m3

```
wt  CCTGCCCCCGCCCCAGCAGCACACTGTGGTTTGTACGGCACTGTGGCCACGTCCAAACCACACTGTGGTGTTAGAGCGAGGGTA
1   CCTGCCCCCGCCCCAGCAGCACACTGTGGTTTGTACGGCACTGTGGCCACGTCCAAACCACACTGTGGTGTTAGAGCGAGGGTA
2   CCTGCCCCCGCCCCAGCAGCACACTGTGGTTTGTACGGCACTGTGGCCACGTCCAAACCACACTGTGGTGTTAGAGCGAGGGTA
3   CCTGCCCCCGCCCCAGCAGCACACTGTGGTTTGTACGGCACTGTGGCCACGTCCAAACCACACTGTGGTGTTAGAGCGAGGGTA
4   CCTGCCCCCGCCCCAGCAGCACACTGTGGTTTGTACGGCACTGTGGCCACGTCCAAACCACACTGTGGTGTTAGAGCGAGGGTA
5   CCTGCCCCCGCCCCAGCAGCACACTGTGGTTTGTACGGCACTGTGGCCACGTCCAAACCACACTGTGGTGTTAGAGCGAGGGTA
6   CCTGCCCCCGCCCCAGCAGCACACTGTGGTTTGTACGGCACTGTGGCCACGTCCAAACCACACTGTGGTGTTAGAGCGAGGGTA
7   CCTGCCCCCGCCCCAGCAGCACACTGTGGTTTGTACGGCACTGTGGCCACGTCCAAACCACACTGTGGTGTTAGAGCGAGGGTA
8   CCTGCCCCCGCCCCAGCAGCACACTGTGGTTTGTACGGCACTGTGGCCACGTCCAAACCACACTGTGGTGTTAGAGCGAGGGTA
9   CCTGCCCCCGCCCCAGCAGCACACTGTGGTTTGTACGGCACTGTGGCCACGTCCAAACCACACTGTGGTGTTAGAGCGAGGGTA
10  CCTGCCCCCGCCCCAGCAGCACACTGTGGTTTGTACGGCACTGTGGCCACGTCCAAACCACACTGTGGTGTTAGAGCGAGGGTA
    ************************************************************************************
```

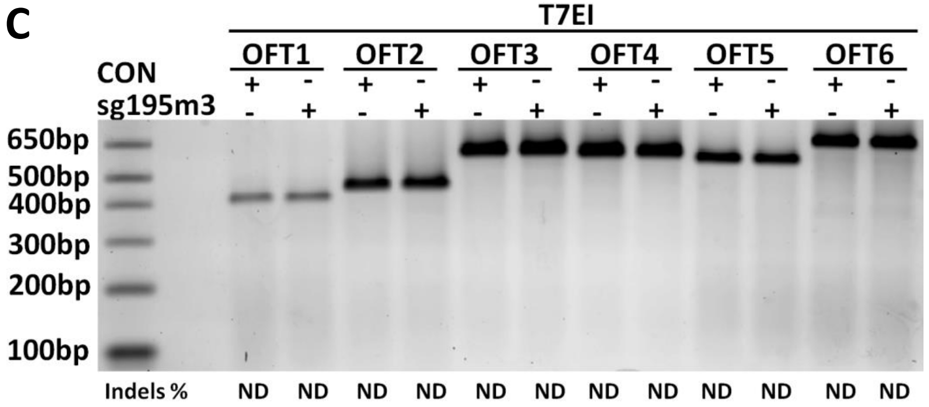

Supplementary Figure S5. Gene editing of the miR-195a locus using sgRNA195 m3. A. Minimum free energy structure of the miR-195a stem loop following sg195m3 editing as assessed by the structure prediction software RNAfold. The base-pairing probability, as indication of structural remodelling or dynamics, was used for color-coding. **B. Clustal alignment of Sanger sequencing of the miR-497a stem loop locus following sg195m3 editing in VSMCs.** Wt: unedited cells, 1-10 randomly picked individual colonies harboring genomic PCR amplicons from sg195m2 edited VSMCs. P3, P4: Genomic PCR primers**. C. Sg195 m3 OFFTARGETS.** T7EI assessment of gene editing in the loci of putative OFFTARGET sites for sg195 m3 as predicted by the CRISPR design tool (Zhang Lab, MIT) and CRISPR Finder (Welcome Trust Sanger Institute). OFT; OFFTARGET, ND: Non Detectable.

Supplementary Figure S6

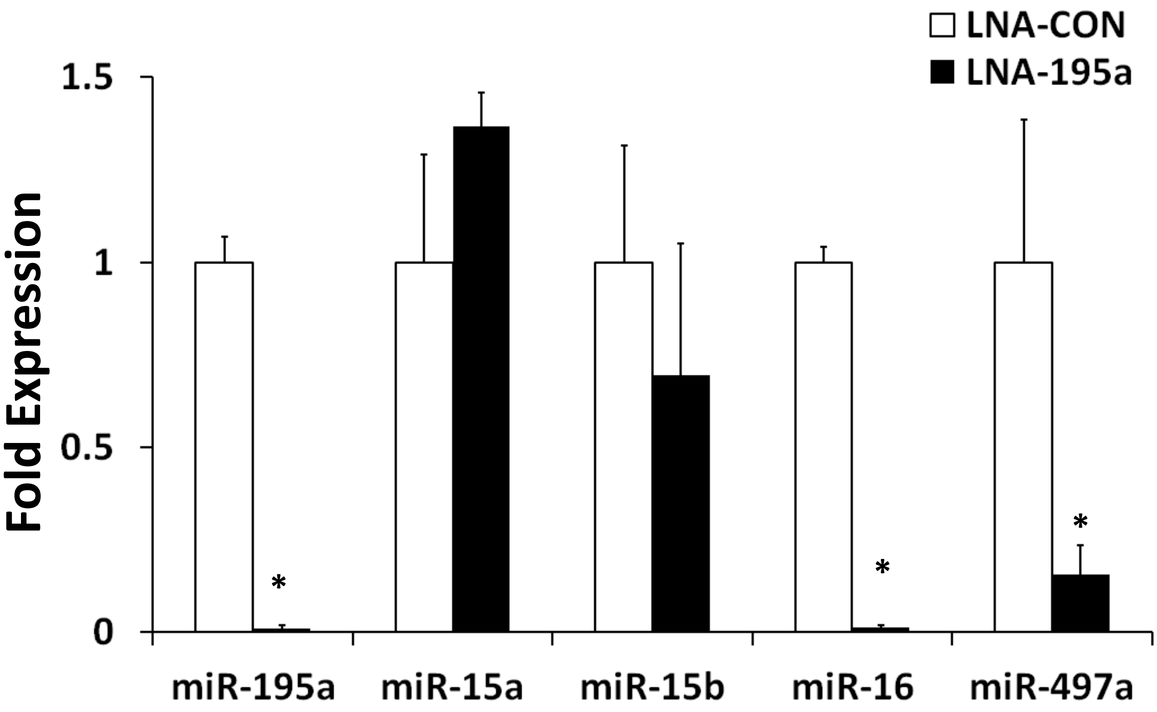

Supplementary Figure S6. QPCR quantification of miRNA expression following miR-195 inhibition. U6 was used as a normalization control. LNA-CON: control LNA oligonucleotide, LNA-195a: LNA anti-miR targeting miR-195a. N=3, * p<0.05

Supplementary Figure S7

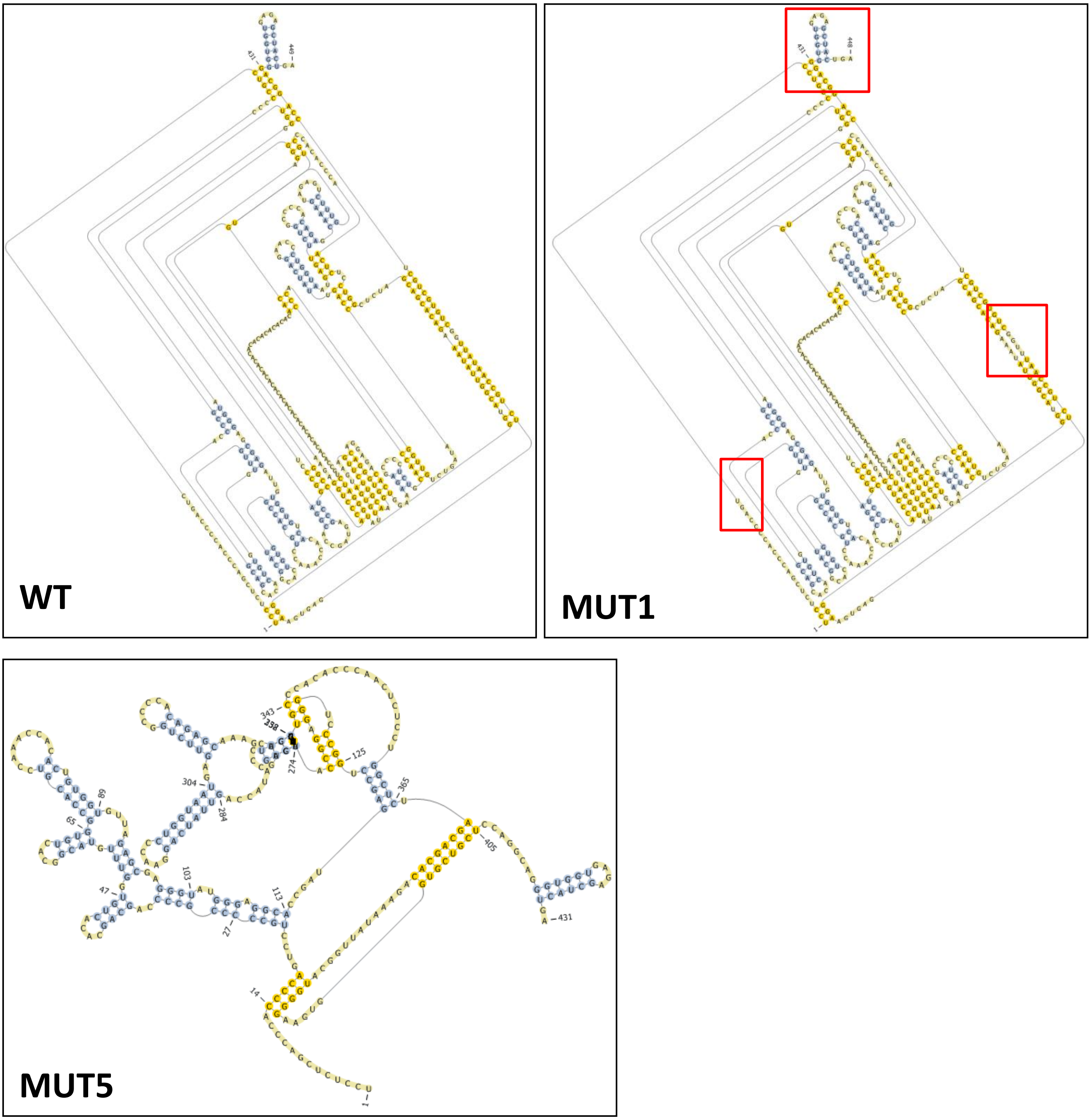

Supplementary Figure S7. Secondary structures with pseudoknots. Generated by vsfold5 (Chiba Institute of Technology, Japan): http://www.rna.it-chiba.ac.jp/~vsfold/vsfold5/ (Settings: 37°C temperature; Kuhn length 6; Jacobson-Stockmayer gamma = 1.75; contiguous stems = 6). WT: unedited cells, MUT1: mutated cells harbouring a 1 nt deletion in miR-195a stem loop, MUT5: mutated cells harbouring a 18nt deletion in the miR-195a stem loop. Red rectangulars in MUT1 highlight differences from WT.

Supplementary Figure S8

WT, L=449

SS without pseudoknots:

..(((((.(((...((((....(((((.(((...((((.((((.(((((((((.(((((......)))..)).))))))))).)))).))))...))).))))))))((((........))))((((......))))((((((((.................
...............................))))))))).((((((........)))))).(((((...((((((((((..((((...))))((((((....))))))......)))))(((((((...)))))))....((((((......)))))).......
.))))))....))))).....((((((((((....((((((((((.((((((((((.((.........)))))))))))))..))))))))))))))).))))))))))))).......

SS with pseudoknots:

[[[..................(((((...((((..((((....)))).((((.((((....))))(((((((.........)).)))))....))))..)))).(((.((((........))))((((..[[[.))))(((((((....[[[[.............
............................)))))))..(((((....[[[[))))).((((]]]]......))))....(((.....))).(((((]]].)))))........((((.((((((.....))))))..((((((((......)))).(((((......
))))))]]]].........))))..))))......((((((((.((((((((((.(([]]......))))))))))))..))))))))).))))))))((((((....))))))..

>miR-497 nt pos: 21 – 104 atom pos: 627-3318

>miR-195a-WT nt pos: 346 – 439 atom pos: 11068-14088

MUT1, L=448

SS without pseudoknots:

..(((((.(((...((((....(((((.(((...((((.((((.(((((((((.(((((......)))..)).))))))))).)))).))))...))).))))))))((((........))))((((......))))((((((((.................
...............................))))))))).((((((........)))))).(((((...((((((((((..((((...))))((((((....))))))......)))))(((((((...)))))))....((((((......))))))......
.))))))....)))))......((((((((((....((((((((((....(((((((.((.........)))))))))....)))))))))))))))).))))))))))))).......

SS with pseudoknots:

[[[.................((((((...((((..((((....)))).((((.((((....))))(((((((.........)).)))))....))))..)))).(((.((((........))))((((..[[[.))))(((((((....[[[[.............
............................)))))))..(((((....[[[[))))).((((]]]]......))))....(((.....))).(((((]]].)))))........((((.((((((.....))))))..((((((((......)))).(((((......
))))))]]]].........))))..))))......(((((((((....(((((((.(([]]......)))))))))....))))))))).)))))))))(((((....)))))...

>miR-497 nt pos: 21 – 104 atom pos: 627-3318

>miR-195-MUT1 nt pos: 346 – 438 atom pos: 11068-14055

MUT5, L= 431

SS without pseudoknots:

..((((((..((((((...((((((((.(((...((((.((((.(((((((((.(((((......)))..)).))))))))).)))).))))...))).))))).)))((((........))))((((......))))..(((((((.((((.(((......
......................((((((.(((......)))..)))).))..((((((....((((..........))))...))))))..((((((.....(((((...(((((((.....)))))))...)))))......((((((......))))))...)))
)))......((.((((...((((.....((((((.....))).)))......))))..)))).))))).)))).))))))))..))))))))))))).......

SS with pseudoknots:

.............(((((....(((((((((((..((((....)))).((((.((((....))))(((((((.........)).)))))....))))..))))..))).))))......(((((.((((..[[[.)))).((((((....[[[[[[...........
...............................))))))...(((((....[[[[))))).((((]]]]......))))....(((.....)))..((((]]].)))).((((......((((((((.....))))))))..((((((......))))))....)))).
....]]]]]].................))))).[[[[[[[[.............))))).....]]]]]]]]........((((((....))))))..

>miR-497 nt pos: 21 - 104 atom pos: 627 - 3318

>miR-195-MUT5 nt pos: 346 – 421 atom pos: 11068 - 13516

Supplementary Figure S8. The optimal secondary structure in dot-bracket notation. Data were generated by vsfold5 (Chiba Institute of Technology, Japan): http://www.rna.it-chiba.ac.jp/~vsfold/vsfold5/ (Settings: 37°C temperature; Kuhn length 6; Jacobson-Stockmayer gamma = 1.75; contiguous stems = 6). WT: unedited cells, MUT1: mutated cells harbouring a 1 nt deletion in miR-195a stem loop, MUT5: mutated cells harbouring a 18nt deletion in the miR-195a stem loop. L: length, atom pos: atomic position. A base pair between base i and j is represented by a '(' at position i and a ')' at position j, unpaired bases are represented by dots (Vienna RNA package). Grouping of base positions are represented as [ ].

Supplementary Figure S9

Supplementary Figure S9. **Reconstruction movie of the tertiary structure of the primiR-497~195.** WT: unedited cluster, MUT1: mutated cluster harbouring a 1 nt deletion in miR-195a stem loop, MUT5: mutated cells harbouring a 18nt deletion in the miR-195a stem loop.

https://nms.kcl.ac.uk/kks/projects/SREP-17-12031.php

Username: cluster
Password: 195497

Supplementary Figure S10

A

```
CLUSTAL W (1.83) multiple sequence alignment

miR-143          --UGAGAUGAAGCACUGUAGCUC
miR-145a         GUCCAGUUUUCCCAGGAAUCCCU
                     ** *    **       *
```

B

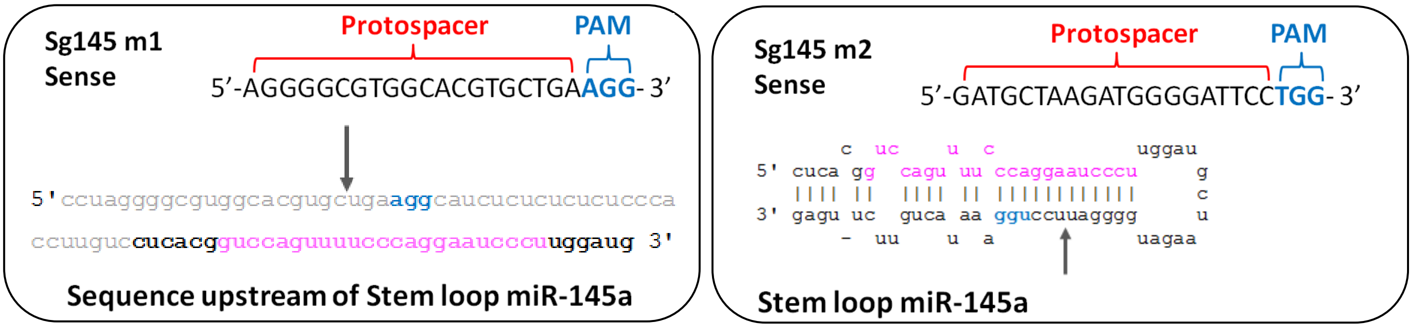

C

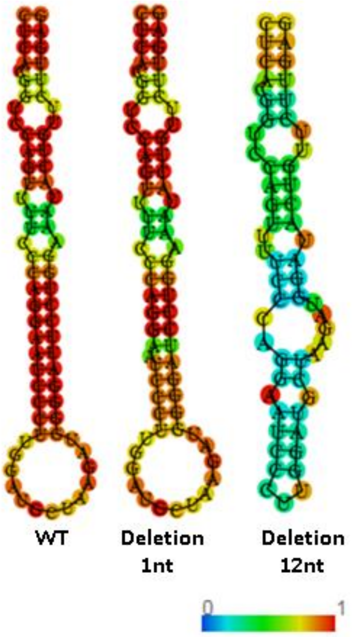

Supplementary Figure S10. A. Sequence Alignment. The T-Coffee multiple sequence alignment program was used to compare the miRNAs located in the mouse miR-143~145 cluster. **B. Schematic representation of sg145 m1 and sg145 m2.** The DNA sequence corresponding to the transcript annotated in miRBase v21 as stem loop miRNA and upstream sequences were used as input to the CRISPR Design tool. PAM sequence is highlighted in blue. The mature miRNA sequence is highlighted in purple and the arrow indicates the site of mutations **C. Minimum free energy structure of the miR-145a stem loop as assessed by the structure prediction software RNAfold.** The base-pairing probability, as indication of structural remodelling or dynamics, was used for color-coding.

Supplementary Figure S11

MiR-143 genomic locus (P5+P6)
Editing: sg145m1

```
wt CCTGAGGTGCAGTGCTGCATCTCTGGTCAGTTGGGAGTCTGAGATGAAGCACTGTAGCTCAGG
1  CCTGAGGTGCAGTGCTGCATCTCTGGTCAGTTGGGAGTCTGAGATGAAGCACTGTAGCTCAGG
2  CCTGAGGTGCAGTGCTGCATCTCTGGTCAGTTGGGAGTCTGAGATGAAGCACTGTAGCTCAGG
3  CCTGAGGTGCAGTGCTGCATCTCTGGTCAGTTGGGAGTCTGAGATGAAGCACTGTAGCTCAGG
4  CCTGAGGTGCAGTGCTGCATCTCTGGTCAGTTGGGAGTCTGAGATGAAGCACTGTAGCTCAGG
5  CCTGAGGTGCAGTGCTGCATCTCTGGTCAGTTGGGAGTCTGAGATGAAGCACTGTAGCTCAGG
6  CCTGAGGTGCAGTGCTGCATCTCTGGTCAGTTGGGAGTCTGAGATGAAGCACTGTAGCTCAGG
7  CCTGAGGTGCAGTGCTGCATCTCTGGTCAGTTGGGAGTCTGAGATGAAGCACTGTAGCTCAGG
8  CCTGAGGTGCAGTGCTGCATCTCTGGTCAGTTGGGAGTCTGAGATGAAGCACTGTAGCTCAGG
9  CCTGAGGTGCAGTGCTGCATCTCTGGTCAGTTGGGAGTCTGAGATGAAGCACTGTAGCTCAGG
10 CCTGAGGTGCAGTGCTGCATCTCTGGTCAGTTGGGAGTCTGAGATGAAGCACTGTAGCTCAGG
   ***************************************************************
```

MiR-143 genomic locus (P5+P6)
Editing: sg145m2

```
wt CCTGAGGTGCAGTGCTGCATCTCTGGTCAGTTGGGAGTCTGAGATGAAGCACTGTAGCTCAGG
1  CCTGAGGTGCAGTGCTGCATCTCTGGTCAGTTGGGAGTCTGAGATGAAGCACTGTAGCTCAGG
2  CCTGAGGTGCAGTGCTGCATCTCTGGTCAGTTGGGAGTCTGAGATGAAGCACTGTAGCTCAGG
3  CCTGAGGTGCAGTGCTGCATCTCTGGTCAGTTGGGAGTCTGAGATGAAGCACTGTAGCTCAGG
4  CCTGAGGTGCAGTGCTGCATCTCTGGTCAGTTGGGAGTCTGAGATGAAGCACTGTAGCTCAGG
5  CCTGAGGTGCAGTGCTGCATCTCTGGTCAGTTGGGAGTCTGAGATGAAGCACTGTAGCTCAGG
6  CCTGAGGTGCAGTGCTGCATCTCTGGTCAGTTGGGAGTCTGAGATGAAGCACTGTAGCTCAGG
7  CCTGAGGTGCAGTGCTGCATCTCTGGTCAGTTGGGAGTCTGAGATGAAGCACTGTAGCTCAGG
8  CCTGAGGTGCAGTGCTGCATCTCTGGTCAGTTGGGAGTCTGAGATGAAGCACTGTAGCTCAGG
9  CCTGAGGTGCAGTGCTGCATCTCTGGTCAGTTGGGAGTCTGAGATGAAGCACTGTAGCTCAGG
10 CCTGAGGTGCAGTGCTGCATCTCTGGTCAGTTGGGAGTCTGAGATGAAGCACTGTAGCTCAGG
   ***************************************************************
```

Supplementary Figure S11. Clustal alignment of Sanger sequencing of the miR-143 stem loop locus following sg145m1 and sg145m2 editing in VSMCs. Wt: unedited cells, 1-10 randomly picked individual colonies harboring genomic PCR amplicons from edited VSMCs. P5, P6: Genomic PCR primers.

Supplementary Figure S12

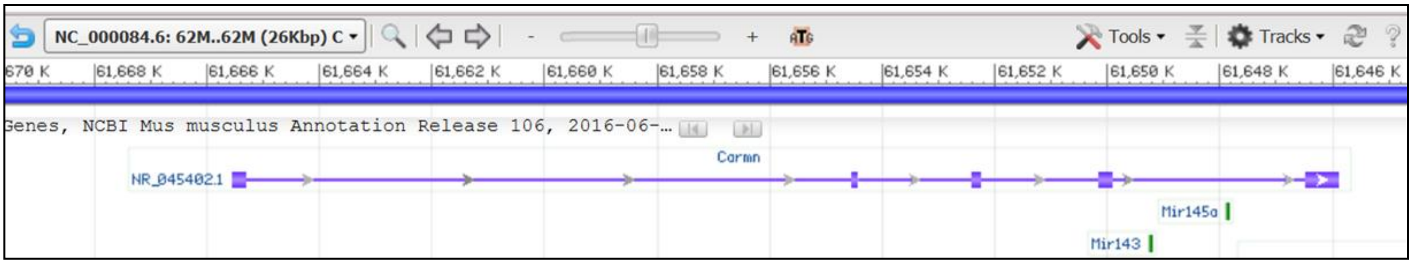

Supplementary Figure S12. Genomic organization of the Carmn locus. Snapshot obtained from NCBI database (https://www.ncbi.nlm.nih.gov/gene/328968) depicting the genomic locus of Carmn (cardiac mesoderm enhancer-associated non-coding RNA) and miR-143~145 cluster.

Supplementary Figure S13

A

```
CLUSTAL W (1.83) multiple sequence alignment

miR-17          CAAAGUGCUUAC-AGUGCA--GGUAG
miR-18a         UAAGGUGCAUCU-AGUGCA--GAUAG
miR-19a         U---GUGCAAAUCUAUGCAAAACUGA
miR-19b         U---GUGCAAAUCCAUGCAAAACUGA
miR-20a         UAAAGUGCUUAU-AGUGCA--GGUAG
miR-92a         UAUU--GCACUU-GUCCCG--GCCUG
                          **          *
```

B

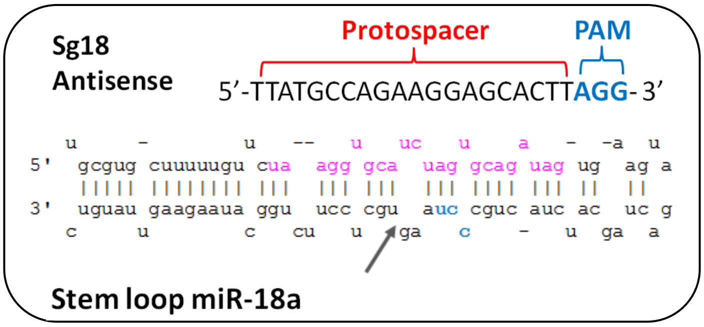

C

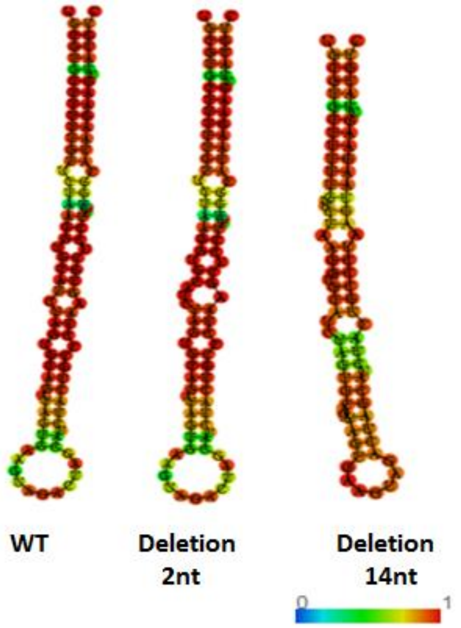

Supplementary Figure S13. A. Sequence Alignment. The T-Coffee multiple sequence alignment program was used to compare the miRNAs residing in the mouse miR-17~92 cluster. **B. Schematic representation of sg18.** The DNA sequence corresponding to the transcript annotated in miRBase v21 as stem loop miRNA was used as input sequence to the CRISPR Design tool. PAM sequence is highlighted in blue. The mature miRNA sequence is highlighted in purple and arrow indicated the site of mutations induced by random indels following editing of the genomic locus in mutant cells. **C. Minimum free energy structure of the miR-18a stem loop as assessed by the structure prediction software RNAfold.** The base-pairing probability, as indication of structural remodelling or dynamics, was used for color-coding.

Supplementary Figure S14

MiR-17 genomic locus (P9+P10)
Editing: sg18

```
wt GTCAGAATAATGTCAAAGTGCTTACAGTGCAGGTAGTGATGTGTGCATCTACTGCAGTGAGGGCACTTGTAGCATTATGCTGAC
1  GTCAGAATAATGTCAAAGTGCTTACAGTGCAGGTAGTGATGTGTGCATCTACTGCAGTGAGGGCACTTGTAGCATTATGCTGAC
2  GTCAGAATAATGTCAAAGTGCTTACAGTGCAGGTAGTGATGTGTGCATCTACTGCAGTGAGGGCACTTGTAGCATTATGCTGAC
3  GTCAGAATAATGTCAAAGTGCTTACAGTGCAGGTAGTGATGTGTGCATCTACTGCAGTGAGGGCACTTGTAGCATTATGCTGAC
4  GTCAGAATAATGTCAAAGTGCTTACAGTGCAGGTAGTGATGTGTGCATCTACTGCAGTGAGGGCACTTGTAGCATTATGCTGAC
5  GTCAGAATAATGTCAAAGTGCTTACAGTGCAGGTAGTGATGTGTGCATCTACTGCAGTGAGGGCACTTGTAGCATTATGCTGAC
6  GTCAGAATAATGTCAAAGTGCTTACAGTGCAGGTAGTGATGTGTGCATCTACTGCAGTGAGGGCACTTGTAGCATTATGCTGAC
7  GTCAGAATAATGTCAAAGTGCTTACAGTGCAGGTAGTGATGTGTGCATCTACTGCAGTGAGGGCACTTGTAGCATTATGCTGAC
8  GTCAGAATAATGTCAAAGTGCTTACAGTGCAGGTAGTGATGTGTGCATCTACTGCAGTGAGGGCACTTGTAGCATTATGCTGAC
   ************************************************************************************
```

MiR-19a genomic locus (P9+P10)
Editing: sg18

```
wt GCAGCCCTCTGTTAGTTTTGCATAGTTGCACTACAAGAAGAATGTAGTTGTGCAAATCTATGCAAAACTGATGGTGGCCTGC
1  GCAGCCCTCTGTTAGTTTTGCATAGTTGCACTACAAGAAGAATGTAGTTGTGCAAATCTATGCAAAACTGATGGTGGCCTGC
2  GCAGCCCTCTGTTAGTTTTGCATAGTTGCACTACAAGAAGAATGTAGTTGTGCAAATCTATGCAAAACTGATGGTGGCCTGC
3  GCAGCCCTCTGTTAGTTTTGCATAGTTGCACTACAAGAAGAATGTAGTTGTGCAAATCTATGCAAAACTGATGGTGGCCTGC
4  GCAGCCCTCTGTTAGTTTTGCATAGTTGCACTACAAGAAGAATGTAGTTGTGCAAATCTATGCAAAACTGATGGTGGCCTGC
5  GCAGCCCTCTGTTAGTTTTGCATAGTTGCACTACAAGAAGAATGTAGTTGTGCAAATCTATGCAAAACTGATGGTGGCCTGC
6  GCAGCCCTCTGTTAGTTTTGCATAGTTGCACTACAAGAAGAATGTAGTTGTGCAAATCTATGCAAAACTGATGGTGGCCTGC
7  GCAGCCCTCTGTTAGTTTTGCATAGTTGCACTACAAGAAGAATGTAGTTGTGCAAATCTATGCAAAACTGATGGTGGCCTGC
8  GCAGCCCTCTGTTAGTTTTGCATAGTTGCACTACAAGAAGAATGTAGTTGTGCAAATCTATGCAAAACTGATGGTGGCCTGC
   **********************************************************************************
```

MiR-20a genomic locus (P9+P10)
Editing: sg18

```
wt GTGTGATGTGACAGCTTCTGTAGCACTAAAGTGCTTATAGTGCAGGTAGTGTGTAGCCATCTACTGCATTACGAGCACTTAAAGTACTGCCAGCTGTAGAACTCCAG
1  GTGTGATGTGACAGCTTCTGTAGCACTAAAGTGCTTATAGTGCAGGTAGTGTGTAGCCATCTACTGCATTACGAGCACTTAAAGTACTGCCAGCTGTAGAACTCCAG
2  GTGTGATGTGACAGCTTCTGTAGCACTAAAGTGCTTATAGTGCAGGTAGTGTGTAGCCATCTACTGCATTACGAGCACTTAAAGTACTGCCAGCTGTAGAACTCCAG
3  GTGTGATGTGACAGCTTCTGTAGCACTAAAGTGCTTATAGTGCAGGTAGTGTGTAGCCATCTACTGCATTACGAGCACTTAAAGTACTGCCAGCTGTAGAACTCCAG
4  GTGTGATGTGACAGCTTCTGTAGCACTAAAGTGCTTATAGTGCAGGTAGTGTGTAGCCATCTACTGCATTACGAGCACTTAAAGTACTGCCAGCTGTAGAACTCCAG
5  GTGTGATGTGACAGCTTCTGTAGCACTAAAGTGCTTATAGTGCAGGTAGTGTGTAGCCATCTACTGCATTACGAGCACTTAAAGTACTGCCAGCTGTAGAACTCCAG
6  GTGTGATGTGACAGCTTCTGTAGCACTAAAGTGCTTATAGTGCAGGTAGTGTGTAGCCATCTACTGCATTACGAGCACTTAAAGTACTGCCAGCTGTAGAACTCCAG
7  GTGTGATGTGACAGCTTCTGTAGCACTAAAGTGCTTATAGTGCAGGTAGTGTGTAGCCATCTACTGCATTACGAGCACTTAAAGTACTGCCAGCTGTAGAACTCCAG
8  GTGTGATGTGACAGCTTCTGTAGCACTAAAGTGCTTATAGTGCAGGTAGTGTGTAGCCATCTACTGCATTACGAGCACTTAAAGTACTGCCAGCTGTAGAACTCCAG
9  GTGTGATGTGACAGCTTCTGTAGCACTAAAGTGCTTATAGTGCAGGTAGTGTGTAGCCATCTACTGCATTACGAGCACTTAAAGTACTGCCAGCTGTAGAACTCCAG
10 GTGTGATGTGACAGCTTCTGTAGCACTAAAGTGCTTATAGTGCAGGTAGTGTGTAGCCATCTACTGCATTACGAGCACTTAAAGTACTGCCAGCTGTAGAACTCCAG
   ***********************************************************************************************************
```

Supplementary Figure S14. Clustal alignment of Sanger sequencing of miR-17, miR-19a and miR-20a stem loop loci following sg18 editing in VSMCs. Wt: unedited cells, 1-10 randomly picked individual colonies harboring genomic PCR amplicons from edited VSMCs. P9, P10: Genomic PCR primers.

Supplementary Figure S15

A

```
CLUSTAL W (1.83) multiple sequence alignment

miR-106b        UAAAGUGCUGA-CAGUGCAG--AU
miR-25          CAUUGCACUUGUCUCGGUCU--GA
miR-93          CAAAGUGCUGU-UCGUGCAGGUAG
                 *  *  **       *
```

B

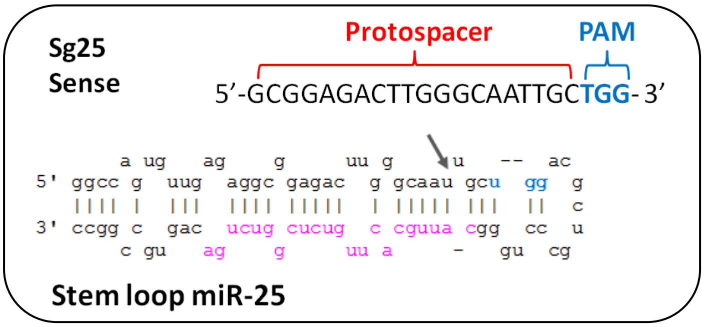

C

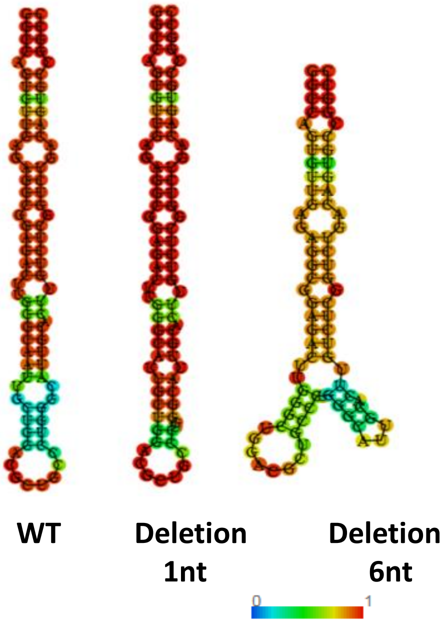

Supplementary Figure S15. A. Sequence Alignment. The T-Coffee multiple sequence alignment program was used to compare the miRNAs residing in the mouse miR-106b~25 cluster. **B. Schematic representation of sg25.** The DNA sequence corresponding to the transcript annotated in miRBase v21 as stem loop miRNA was used as input sequence to the CRISPR Design tool. PAM sequence is highlighted in blue. The mature miRNA sequence is highlighted in purple and the arrow indicates the site of mutations induced by random indels following editing of the genomic locus in mutant cells. **C. Minimum free energy structure of the miR-25 stem loop as assessed by the structure prediction software RNAfold.** The base-pairing probability, as an indication of structural remodelling or dynamics, was used for color-coding.

Supplementary Figure S16

MiR-93 genomic locus (P11+P12)
Editing: sg25

```
wt AGTCATGGGGGCTCCAAAGTGCTGTTCGTGCAGGTAGTGTAATTACCTGACCTACTGCTGAGCTAGCACTTCCCGAGCCCCCAGGACA
1  AGTCATGGGGGCTCCAAAGTGCTGTTCGTGCAGGTAGTGTAATTACCTGACCTACTGCTGAGCTAGCACTTCCCGAGCCCCCAGGACA
2  AGTCATGGGGGCTCCAAAGTGCTGTTCGTGCAGGTAGTGTAATTACCTGACCTACTGCTGAGCTAGCACTTCCCGAGCCCCCAGGACA
3  AGTCATGGGGGCTCCAAAGTGCTGTTCGTGCAGGTAGTGTAATTACCTGACCTACTGCTGAGCTAGCACTTCCCGAGCCCCCAGGACA
4  AGTCATGGGGGCTCCAAAGTGCTGTTCGTGCAGGTAGTGTAATTACCTGACCTACTGCTGAGCTAGCACTTCCCGAGCCCCCAGGACA
5  AGTCATGGGGGCTCCAAAGTGCTGTTCGTGCAGGTAGTGTAATTACCTGACCTACTGCTGAGCTAGCACTTCCCGAGCCCCCAGGACA
6  AGTCATGGGGGCTCCAAAGTGCTGTTCGTGCAGGTAGTGTAATTACCTGACCTACTGCTGAGCTAGCACTTCCCGAGCCCCCAGGACA
7  AGTCATGGGGGCTCCAAAGTGCTGTTCGTGCAGGTAGTGTAATTACCTGACCTACTGCTGAGCTAGCACTTCCCGAGCCCCCAGGACA
8  AGTCATGGGGGCTCCAAAGTGCTGTTCGTGCAGGTAGTGTAATTACCTGACCTACTGCTGAGCTAGCACTTCCCGAGCCCCCAGGACA
9  AGTCATGGGGGCTCCAAAGTGCTGTTCGTGCAGGTAGTGTAATTACCTGACCTACTGCTGAGCTAGCACTTCCCGAGCCCCCAGGACA
10 AGTCATGG-GGCTCCAAAGTGCTGTTCGTGCAGGTAGTGTAATTACCTGACCTACTGCTGAGCTAGCACTTCCCGAGCCCCCAGGACA
   ******** *******************************************************************************
```

MiR-106b genomic locus (P11+P12)
Editing: sg25

```
wt CCTGCTGGGACTAAAGTGCTGACAGTGCAGATAGTGGTCCTCTCTGTGCTACCGCACTGTGGGTACTTGCTGCTCCAGCAGG
1  CCTGCTGGGACTAAAGTGCTGACAGTGCAGATAGTGGTCCTCTCTGTGCTACCGCACTGTGGGTACTTGCTGCTCCAGCAGG
2  CCTGCTGGGACTAAAGTGCTGACAGTGCAGATAGTGGTCCTCTCTGTGCTACCGCACTGTGGGTACTTGCTGCTCCAGCAGG
3  CCTGCTGGGACTAAAGTGCTGACAGTGCAGATAGTGGTCCTCTCTGTGCTACCGCACTGTGGGTACTTGCTGCTCCAGCAGG
4  CCTGCTGGGACTAAAGTGCTGACAGTGCAGATAGTGGTCCTCTCTGTGCTACCGCACTGTGGGTACTTGCTGCTCCAGCAGG
5  CCTGCTGGGACTAAAGTGCTGACAGTGCAGATAGTGGTCCTCTCTGTGCTACCGCACTGTGGGTACTTGCTGCTCCAGCAGG
6  CCTGCTGGGACTAAAGTGCTGACAGTGCAGATAGTGGTCCTCTCTGTGCTACCGCACTGTGGGTACTTGCTGCTCCAGCAGG
7  CCTGCTGGGACTAAAGTGCTGACAGTGCAGATAGTGGTCCTCTCTGTGCTACCGCACTGTGGGTACTTGCTGCTCCAGCAGG
8  CCTGCTGGGACTAAAGTGCTGACAGTGCAGATAGTGGTCCTCTCTGTGCTACCGCACTGTGGGTACTTGCTGCTCCAGCAGG
9  CCTGCTGGGACTAAAGTGCTGACAGTGCAGATAGTGGTCCTCTCTGTGCTACCGCACTGTGGGTACTTGCTGCTCCAGCAGG
10 CCTGCTGGGACTAAAGTGCTGACAGTGCAGATAGTGGTCCTCTCTGTGCTACCGCACTGTGGGTACTTGCTGCTCCAGCAGG
   **********************************************************************************
```

Supplementary Figure S16. Clustal alignment of Sanger sequencing of miR-93 and miR-106b stem loop loci following sg25 editing in VSMCs. Wt: unedited cells, 1-10 randomly picked individual colonies harboring genomic PCR amplicons from edited VSMCs. P11, P12: Genomic PCR primers.

Supplementary Table 1.

miRNA [miRNA family]	MiRBase stem loop accession number	Coordinates (GRCm38)	Mature miRNA Sequence	Cluster
**miR-15a [miR-15]**	MI0000564	chr14: 61632027-61632110 [-]	U**AGCAGCA**CAUAAUGGUUUGUG	**miR-15a~16-1**
**miR-16-1 [miR-15]**	MI0000565	chr14: 61631880-61631972 [-]	U**AGCAGCA**CGUAAAUAUUGGCG	**miR-15a~16-1**
**miR-15b [miR-15]**	MI0000140	chr3: 69009772-69009835 [+]	U**AGCAGCA**CAUCAUGGUUUACA	**miR-15b~16-2**
**miR-16-2 [miR-15]**	MI0000566	chr3: 69009902-69009996 [+]	U**AGCAGCA**CGUAAAUAUUGGCG	**miR-15b~16-2**
**miR-195 [miR-15]**	MI0000237	chr11: 70235042-70235135 [+]	U**AGCAGCA**CAGAAAUAUUGGC	**miR-497~195**
**miR-497a [miR-15]**	MI0004636	chr11: 70234717-70234800 [+]	C**AGCAGCA**CACUGUGGUUUGUA	**miR-497~195**
**miR-497b [miR-140]**	MI0026030	chr11: 70234692-70234816 [-]	C**ACCACAG**UGUGGUUUGGACGUGG	**miR-497~195**
**miR-143 [miR-143]**	MI0000257	chr18: 61649196-61649258 [-]	G**GUGCAGU**GCUGCAUCUCUGG	**miR-143~145**
**miR-145 [miR-145]**	MI0000169	chr18: 61647825-61647894 [-]	G**UCCAGUU**UUCCCAGGAAUCCCU	**miR-143~145**
**miR-17 [miR-17]**	MI0000687	chr14: 115043671-115043754 [+]	C**AAAGUGC**UUACAGUGCAGGUAG	**miR-17~92**
**miR-18a [miR-18]**	MI0000567	chr14: 115043851-115043946 [+]	U**AAGGUGC**AUCUAGUGCAGAUAG	**miR-17~92**
**miR-19a [miR-19]**	MI0000688	chr14: 115044000-115044081 [+]	U**GUGCAAA**UCUAUGCAAAACUGA	**miR-17~92**
**miR-20a [miR-17]**	MI0000568	chr14: 115044157-115044263 [+]	U**AAAGUGC**UUAUAGUGCAGGUAG	**miR-17~92**
**miR-19b-1 [miR-19]**	MI0000718	chr14: 115044305-115044391 [+]	U**GUGCAAA**UCCAUGCAAAACUGA	**miR-17~92**
**miR-92a-1 [miR-25]**	MI0000719	chr14: 115044427-115044506 [+]	U**AUUGCAC**UUGUCCCGGCCUG	**miR-17~92**
**miR-106b [miR-17]**	MI0000407	chr5: 138165737-138165818 [-]	U**AAAGUGC**UGACAGUGCAGAU	**miR-106b~25**
**miR-93 [miR-17]**	MI0000581	chr5: 138165523-138165610 [-]	C**AAAGUGC**UGUUCGUGCAGGUAG	**miR-106b~25**
**miR-25 [miR-25]**	MI0000689	chr5: 138165321-138165404 [-]	C**AUUGCAC**UUGUCUCGGUCUGA	**miR-106b~25**
**miR-106a [miR-17]**	MI0000406	chrX: 52742503-52742567 [-]	C**AAAGUGC**UAACAGUGCAGGUAG	**miR-106a~363**
**miR-18b [miR-18]**	MI0005483	chrX: 52742331-52742413 [-]	U**AAGGUGC**AUCUAGUGCUGUUAG	**miR-106a~363**
**miR-20b [miR-17]**	MI0003536	chrX: 52742113-52742192 [-]	C**AAAGUGC**UCAUAGUGCAGGUAG	**miR-106a~363**
**miR-19b-2 [miR-19]**	MI0000546	chrX: 52741983-52742066 [-]	U**GUGCAAA**UCCAUGCAAAACUGA	**miR-106a~363**
**miR-92a-2 [miR-25]**	MI0000580	chrX: 52741838-52741928 [-]	U**AUUGCAC**UUGUCCCGGCCUG	**miR-106a~363**
**miR-363 [miR-25]**	MI0000765	chrX: 52741693-52741767 [-]	C**AGGUGGA**ACACGAUGCAAUUU	**miR-106a~363**

Supplementary Table 1. Details of miRNA clusters and miRNA families. Data on miRNA clusters were obtained from the MiRBase database. Data on miRNA families were obtained from the Targetscan programme.

Supplementary Table 2.

Assay	Primer	Sequence (5' to 3')	Coordinates (GRCm38)
**IVT**	F 195m2	TAATACGACTCACTATAGGTGGAGCAGCACAGCCAATAT	chr11: 70,235,100-70,235,120
**IVT**	R 195m2	TTCTAGCTCTAAAACATATTGGCTGTGCTGCTCCAC	
**IVT**	F 195m3	TAATACGACTCACTATAGCTCTAGCAGCACAGAAATAT	chr11: 70,235,059-70,235,079
**IVT**	R 195m3	TTCTAGCTCTAAAACATATTTCTGTGCTGCTAGAG	
**IVT**	F 145m1	TAATACGACTCACTATAGAGGGGCGTGGCACGTGCTGA	chr18: 61,647,919-61,647,939
**IVT**	R 145m1	TTCTAGCTCTAAAACTCAGCACGTGCCACGCCCCT	
**IVT**	F 145m2	TAATACGACTCACTATAGGATGCTAAGATGGGGATTCC	chr18: 61,647,840-61,647,860
**IVT**	R 145m2	TTCTAGCTCTAAAACGGAATCCCCATCTTAGCATC	
**IVT**	F 18a	TAATACGACTCACTATAGTTATGCCAGAAGGAGCACTT	chr14: 115,043,915-115,043,935
**IVT**	R 18a	TTCTAGCTCTAAAACAAGTGCTCCTTCTGGCATAA	
**IVT**	F 25	TAATACGACTCACTATAGGCGGAGACTTGGGCAATTGC	chr5: 138,165,367-138,165,387
**IVT**	R 25	TTCTAGCTCTAAAACGCAATTGCCCAAGTCTCCGC	
**GENOMIC PCR**	MiR-195 F (P1)	CACACACACACCGTCTAGGG	chr11: 70,234,878-70,235,407
**GENOMIC PCR**	MiR-195 R (P2)	CTGAGCCTTCCACCTCTGAC	
**GENOMIC PCR**	MiR-497 F (P3)	CCTGTGTCTTCCAGCATTTCTC	chr11: 70,234,360- 70,234,977
**GENOMIC PCR**	MiR-497 R (P4)	GTATCAGACAACCTGGGGGTT	
**GENOMIC PCR**	MiR-143 F (P5)	GTGCTGCGTGCATAAAGAGA	chr18: 61,649,137- 61,649,567
**GENOMIC PCR**	MiR-143 R (P6)	GCTATCCCATGCCAACACTT	
**GENOMIC PCR**	MiR-145 F (P7)	CTTTCCAAGCCACTCAAAGC	chr18: 61,647,566- 61,648,054
**GENOMIC PCR**	MiR-145 R (P8)	GGAGCCGTCTCATAGTCTGG	
**GENOMIC PCR**	MiR-18 F (P9)	CCTGGTCAATGTGAGGCTTT	chr14: 115,043,309- 115,044,386
**GENOMIC PCR**	MiR-18 R (P10)	CCACAGTCAGTTTTGCATGG	
**GENOMIC PCR**	MiR-25 F (P11)	TTCTCCGACTTTCCACTGCT	chr5: 138,164,956-138,165,932
**GENOMIC PCR**	MiR-25 R (P12)	GCCACAAACAGTAGCAGCAA	
**Gene expression**	Carmn F	AATAGACTGGGCCTCCACCT	chr18: 61,651,154- 61,651,267
**Gene expression**	Carmn R	GTTCCTCTCTGGGGCTCTTC	

Supplementary Table 2. Primer sequences. Genomic primers were labelled P1-P12, as depicted on the genomic loci schematics. IVT: *in vitro* transcription.

Supplementary Table 3.

Assay	GENE	OFFTARGET Sequence	Mismatches	Primer Sequence (5' to 3')		Locus
GENOMIC PCR	OFFTARGET1 195m2	AGGAGCAGTGCAGCCAATAT	3MMs [1:9:10]	OFT1 195m2 F	GTGCTGGGATTACAGGCATT	chr17:-3133199
GENOMIC PCR				OFT1 195m2 R	TCCTTGGAATGAGAGGGCTA	
GENOMIC PCR	OFFTARGET2 195m2	AGGAGCAACACAGCCACTAT	3MMs [1:8:17]	OFT2 195m2 F	GCCCCAGAGTGTGTGAAAGT	chr8:-10246601
GENOMIC PCR				OFT2 195m2 R	TCAAGCCTCTGAATGCACAC	
GENOMIC PCR	OFFTARGET3 195m2	ATGAGCAGCACAGCGAATAT	3MMs [1:2:15]	OFT3 195m2 F	TCCACATGGACTGCAGTGTT	chr14:-73777792
GENOMIC PCR				OFT3 195m2 R	GCTCCTGTGTTCAGGTGTGA	
GENOMIC PCR	OFFTARGET4 195m2	TGGAGCACAAAAGCCAATAT	3MMs [8:9:11]	OFT4 195m2 F	ATTCTCGGGGAAATTCCATC	chrX:+72165663
GENOMIC PCR				OFT4 195m2 R	TTGCACTGCTGTCACACAAA	
GENOMIC PCR	OFFTARGET5 195m2	TGGAGCATCACAGCCAACAT	2MMs [8:17]	OFT5 195m2 F	TATCCTCTCGCCTTTGCACT	chr2:+24952183
GENOMIC PCR				OFT5 195m2 R	GCAGAGTGCATCTGTTGGAA	
GENOMIC PCR	OFFTARGET6 195m2	TGCAGCAGCAAAGCCACTAT	3MMs [3:11:17]	OFT6 195m2 F	CGACTTGCAATCACATGGAG	chr13:+35216430
GENOMIC PCR				OFT6 195m2 R	CACCCACTCGGGACCTACTA	
GENOMIC PCR	OFFTARGET7 195m2	AGGATTAGCTCAGCCAATAT	4MMs [1:5:6:10]	OFT7 195m2 F	AGTGCTGGGCATAGGACAAC	chr2:-91834510
GENOMIC PCR				OFT7 195m2 R	CTGCTCTTCCAAAGGTCCTG	
GENOMIC PCR	OFFTARGET1 195m3	CTGTAGCAACACAGAAATAT	2MMs [3:9]	OFT1 195m3 F	TGGGTTTGAAGGTCCTGAAG	chr1:-196773757
GENOMIC PCR				OFT1 195m3 R	ACATCCTTGTTGCCCATGAT	
GENOMIC PCR	OFFTARGET2 195m3	CTCTAGCAGGACAGAAATAA	2MMs [10:20]	OFT2 195m3 F	TCTGCCTCAGCTTTCCAAGT	chr4:+62834971
GENOMIC PCR				OFT2 195m3 R	TGCTCAAGCTACACCCAGTG	
GENOMIC PCR	OFFTARGET3 195m3	TTAAAGCAGCACAGAAATAT	3MMs [1:3:4]	OFT3 195m3 F	TCCCCTGGACTTTGACATTC	chr3:-154152635
GENOMIC PCR				OFT3 195m3 R	AATGCCCTTCCCCAATAATC	
GENOMIC PCR	OFFTARGET4 195m3	AGCTAGCAGCTCAGAAATAT	3MMs [1:2:11]	OFT4 195m3 F	TGGAGAAGGAGGAGGTCTGA	chr15:-76275970
GENOMIC PCR				OFT4 195m3 R	GTGCCTGAACTCTGTGGTGA	
GENOMIC PCR	OFFTARGET5 195m3	GTCTACCAGAACAGAAATAT	3MMs [1:6:10]	OFT5 195m3 F	CCCATTTGCATGGAAAGATT	chr12:+31442719
GENOMIC PCR				OFT5 195m3 R	TGAAATTGGGGAGTGTGTGA	
GENOMIC PCR	OFFTARGET6 195m3	CTCTGTCTGCACAGAAATAT	3MMs [5:6:8]	OFT6 195m3 F	GGGAGACTATTGTGCATGATTTTATT	chr9:-22593762
GENOMIC PCR				OFT6 195m3 R	GGTATTACTGTTTAGGGCCACTCT	

Supplementary Table 3. OFFTARGET Primer sequences. Genomic primers for putative OFFTARGET sites of editing for sg195 m2 and sg195 m3 as predicted by the CRISPR Design tool (Zhang lab, MIT) or the CRISPR Finder (Welcome Trust Sanger Institute- highlighted in blue). The mismatches are shown in red. OFT: OFFTARGET.

ONLINE SUPPLEMENT

CRISPR/Cas9 editing reveals novel mechanisms of clustered microRNA regulation and function

Lazaros Lataniotis[1], Andreas Albrecht[2], Fatma O. Kok[3], Clinton A.L. Monfries[4], Lorena Benedetti[5], Nathan D. Lawson[3], Simon M. Hughes[4], Kathleen Steinhofel[6], Manuel Mayr[1] and Anna Zampetaki[1]

1 King's British Heart Foundation Centre, King's College London, UK.
2 Middlesex University, School of Science and Technology, London, UK.
3 University of Massachusetts Medical School, Department of Molecular, Cell, and Cancer Biology, Worcester, USA.
4 King's College London, Randall Division, London, UK.
5 King's College London, Division of Cancer Studies, London, UK.
6 King's College London, Informatics Department, London, UK.

UNCROPPED GEL IMAGES

Figure 1

Fig1b

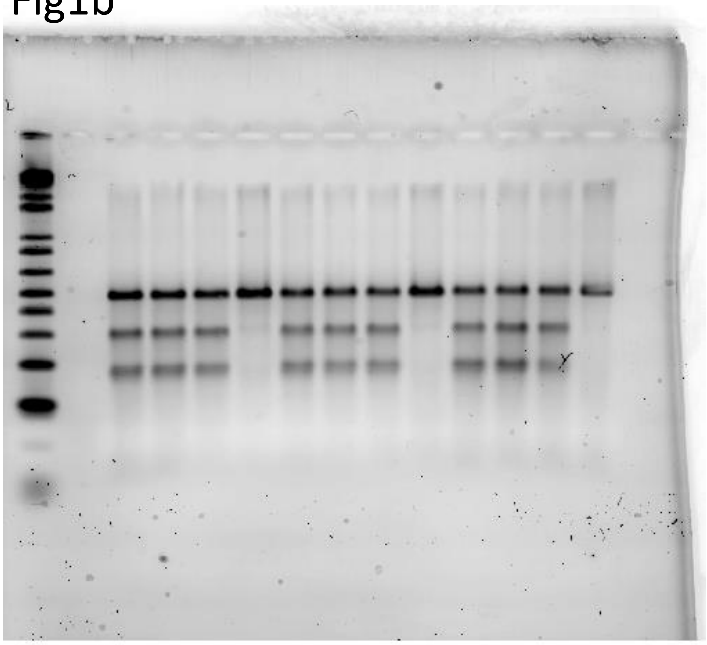

Fig1c

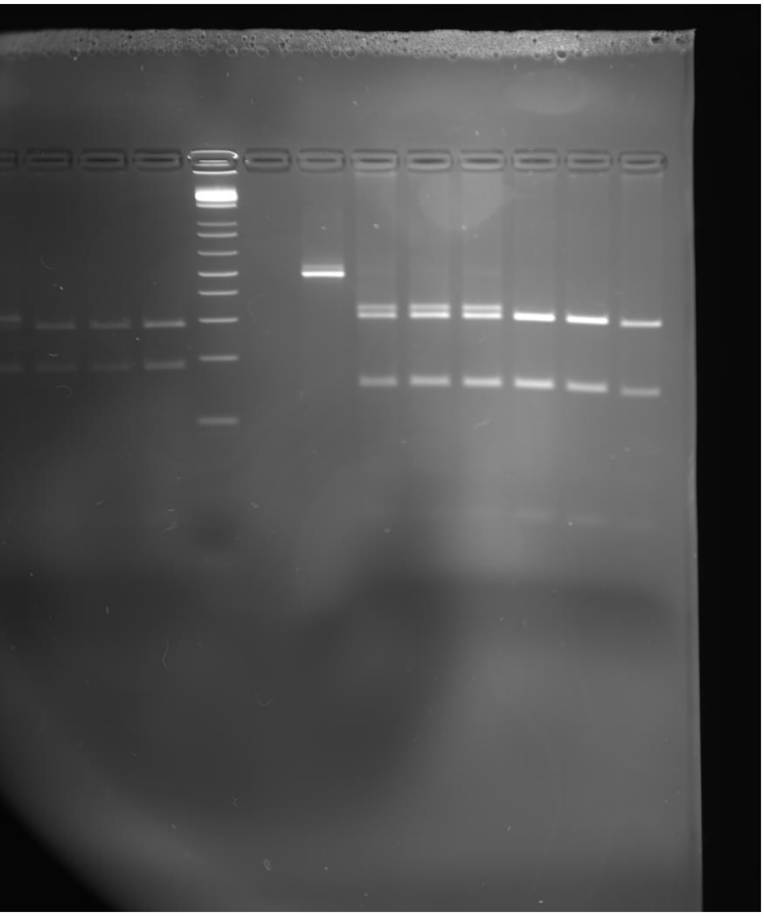

Fig1f

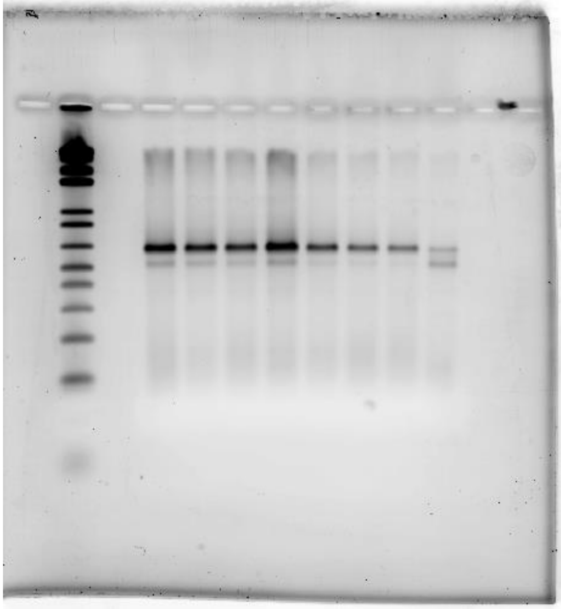

Figure 3

Fig3b

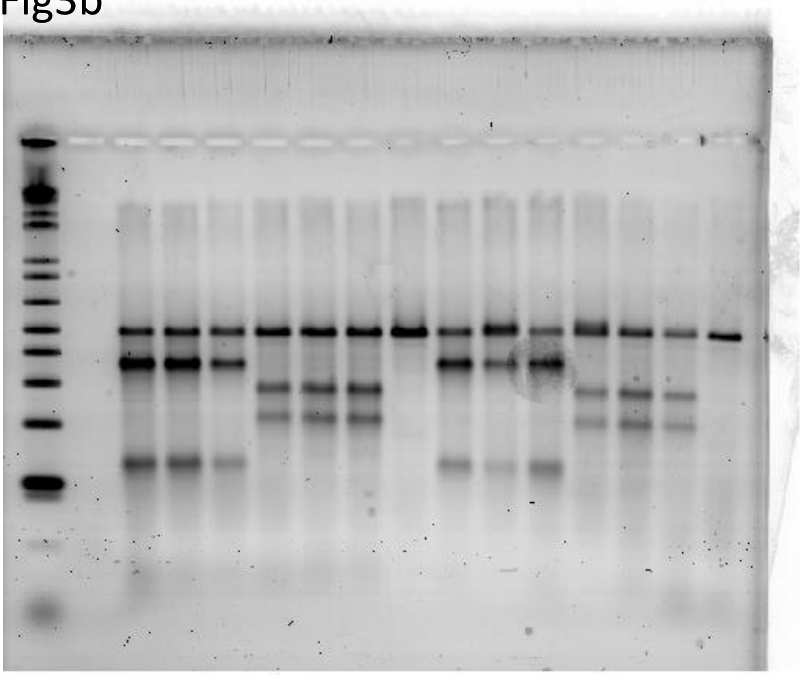

Fig3c

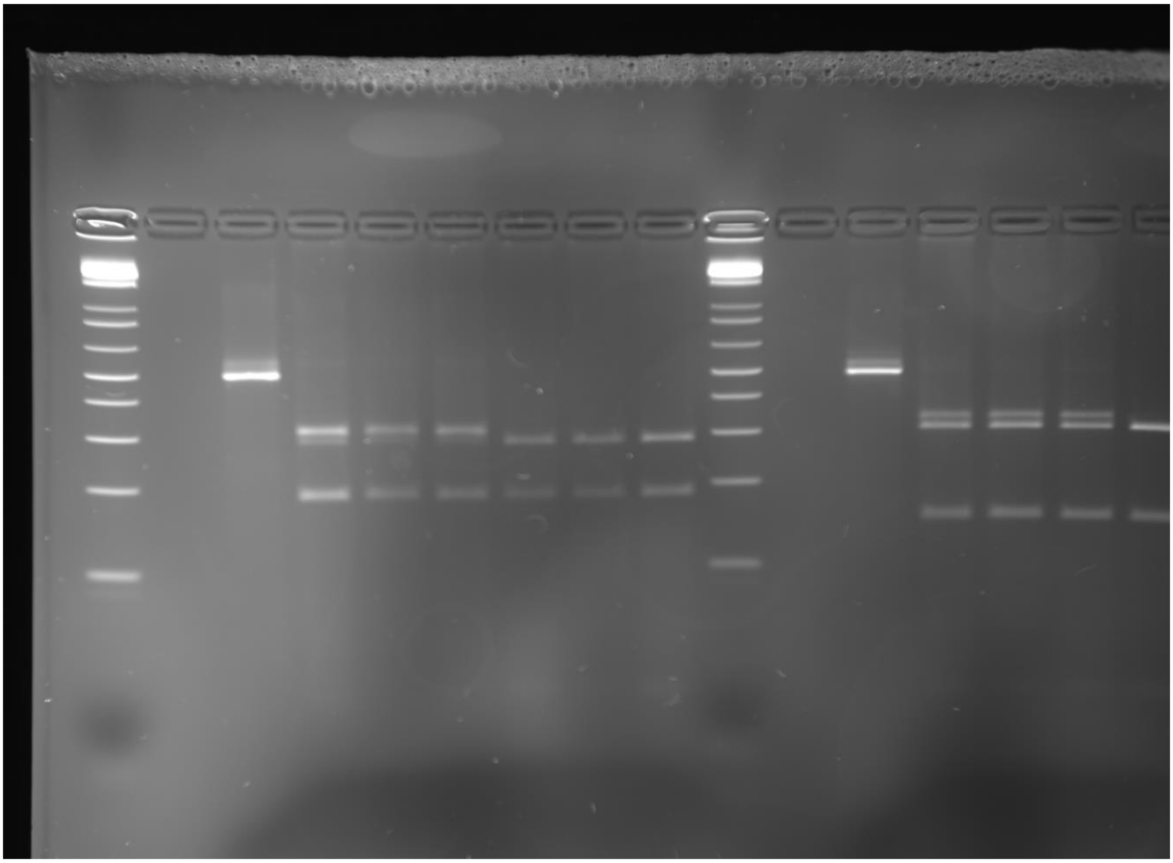

Figure 4

Fig4b

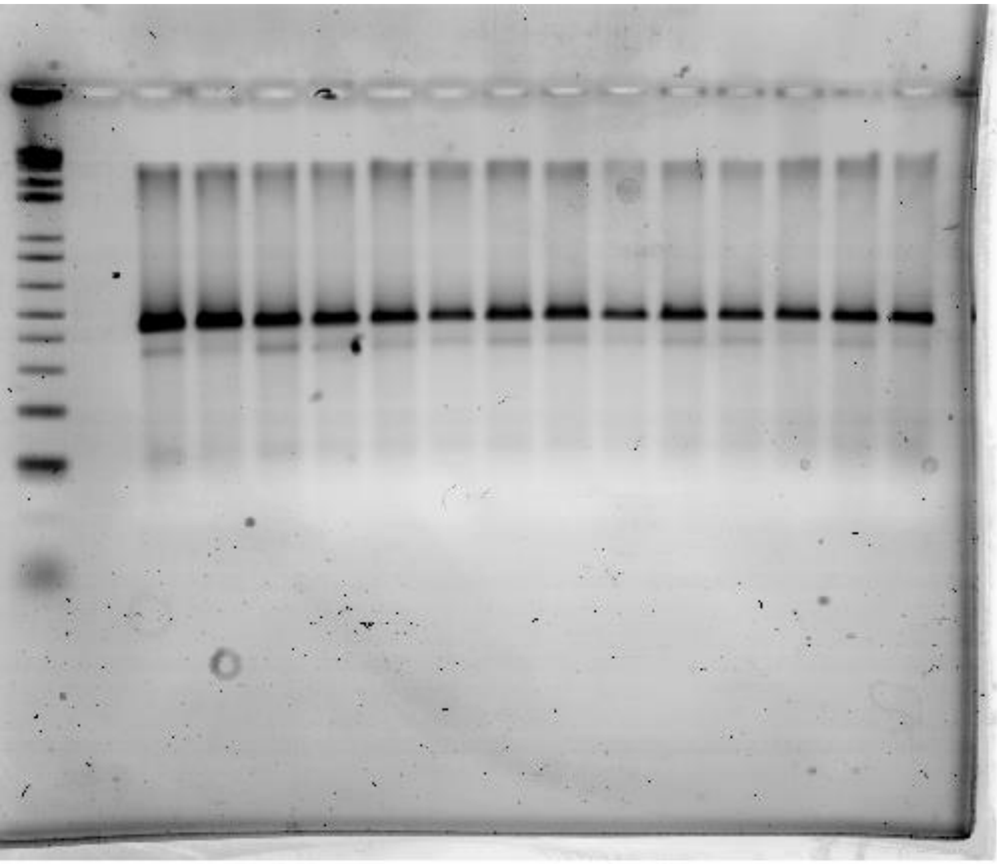

Figure 5

Fig5b

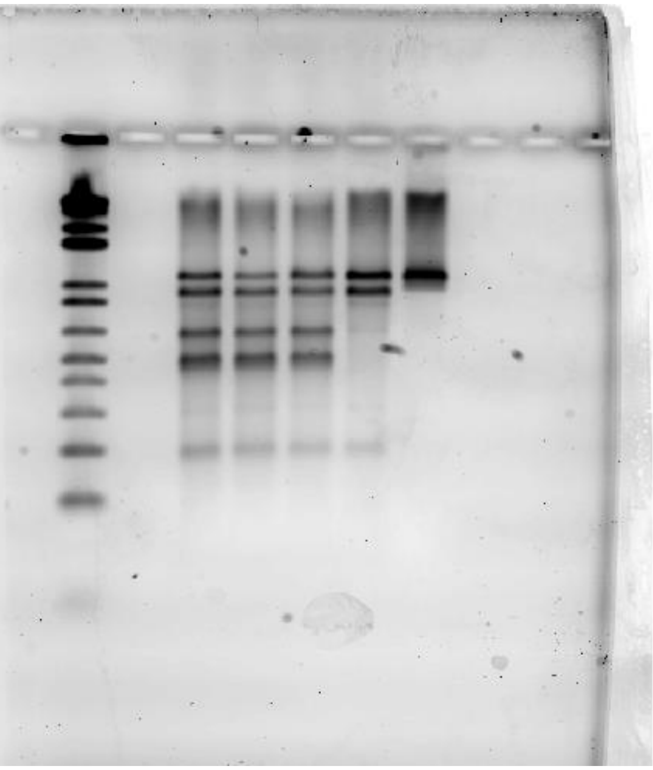

Fig5c

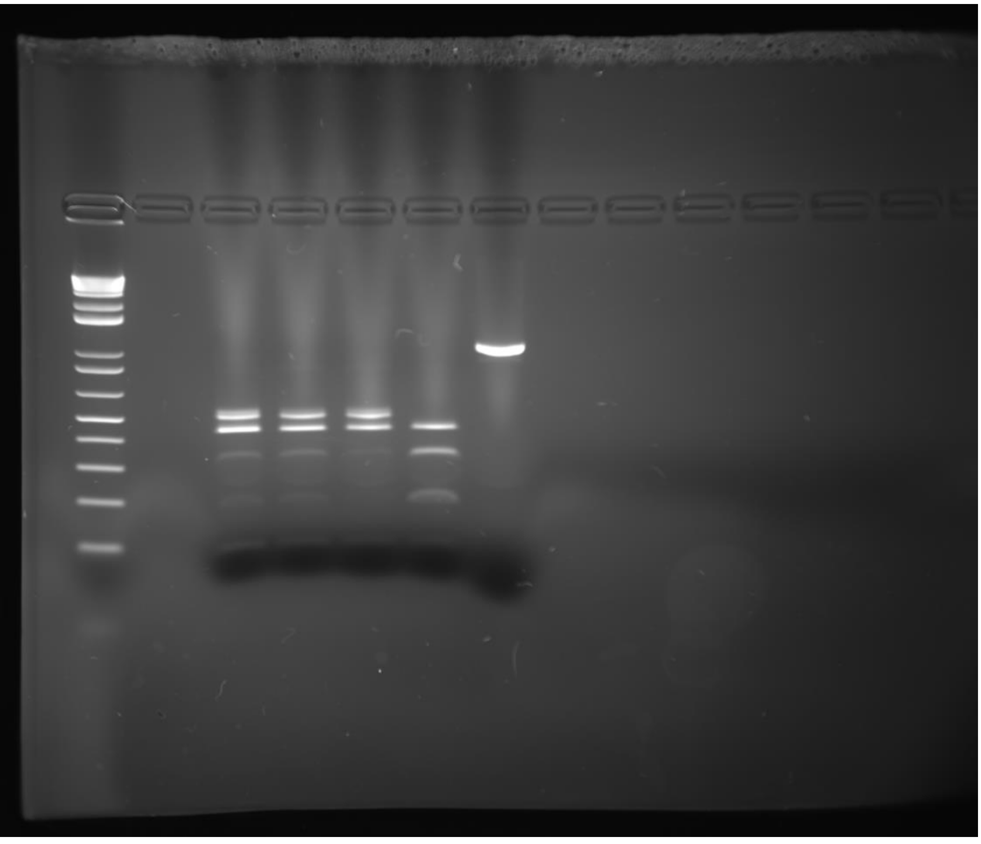

Figure 6

Fig6b

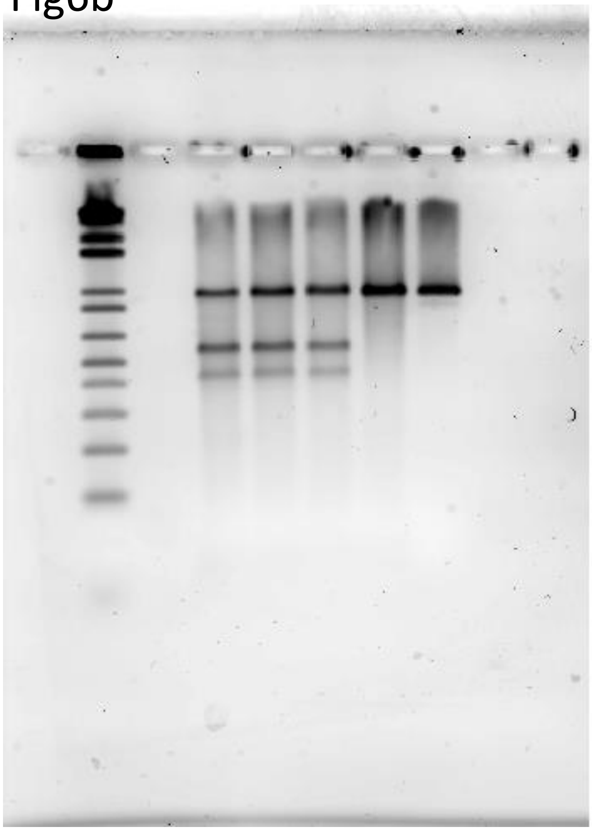

Fig6c

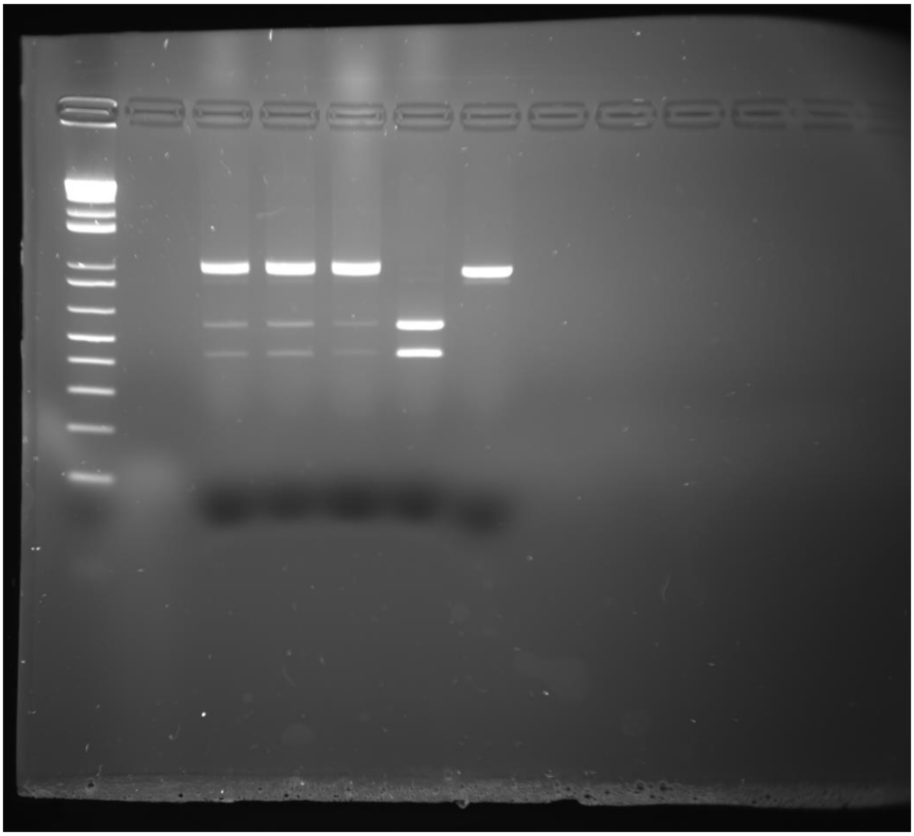

Supplementary Figure 3

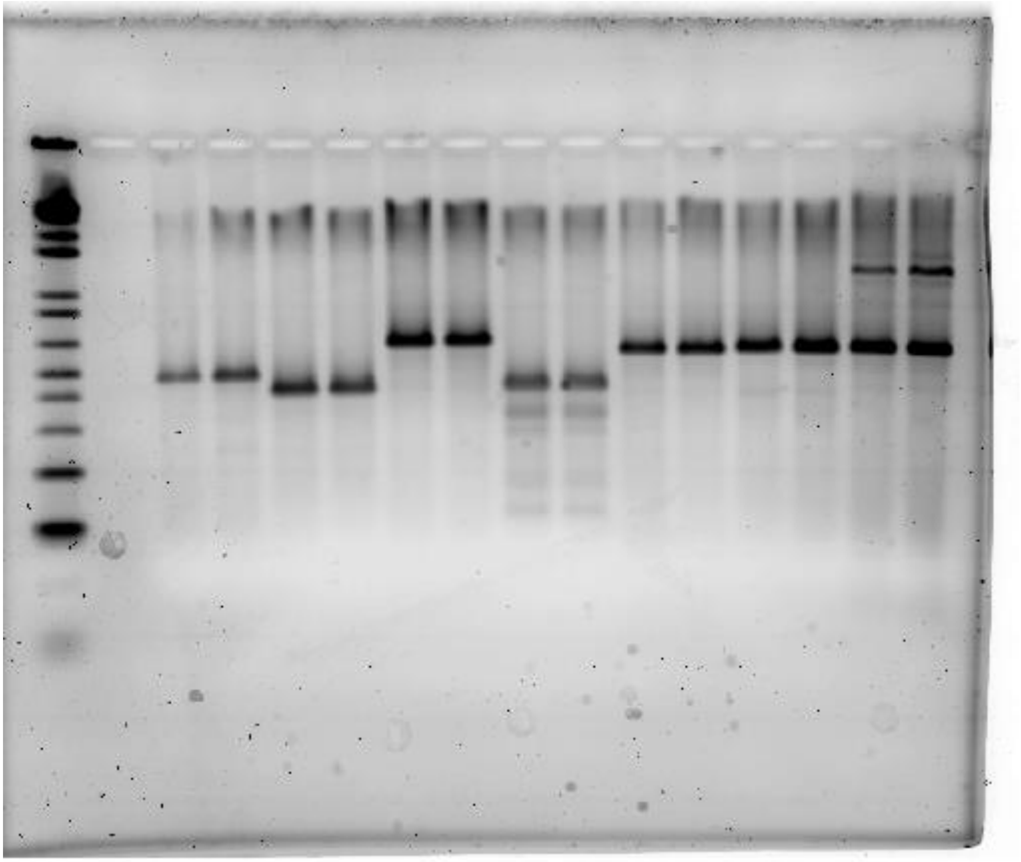

Supplementary Figure 4

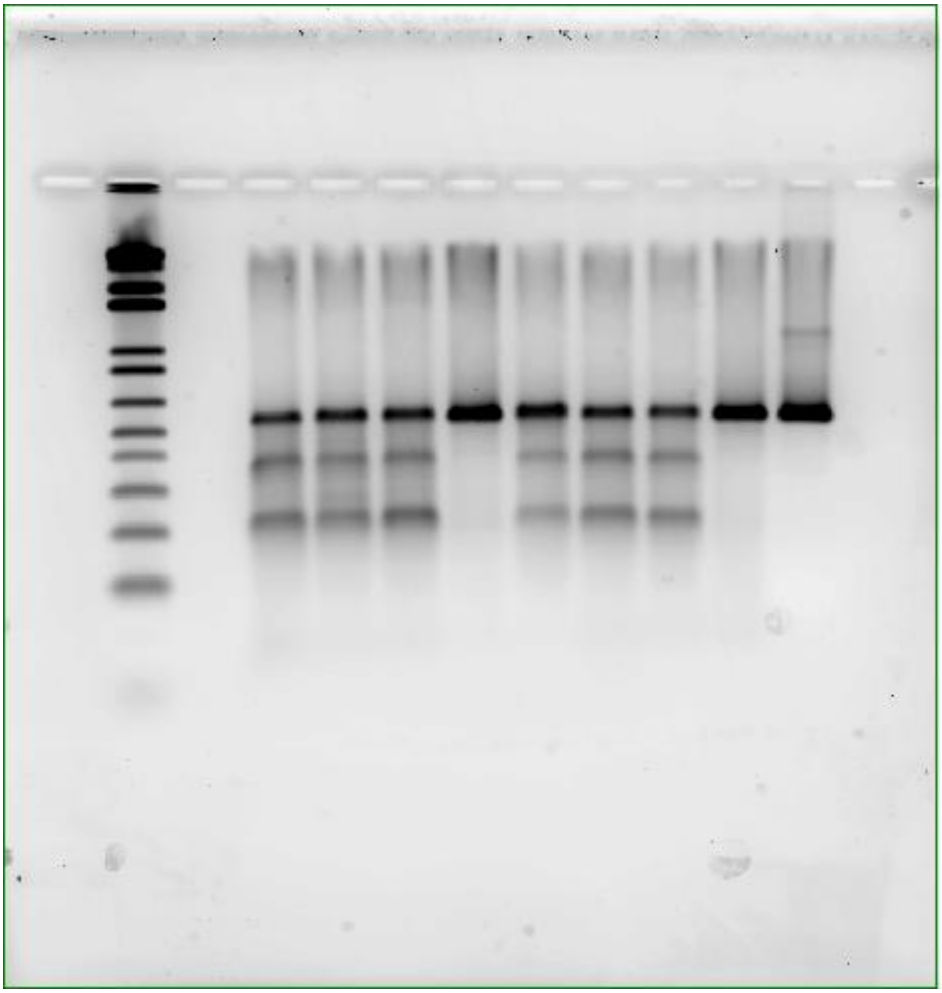

Supplementary Figure 5

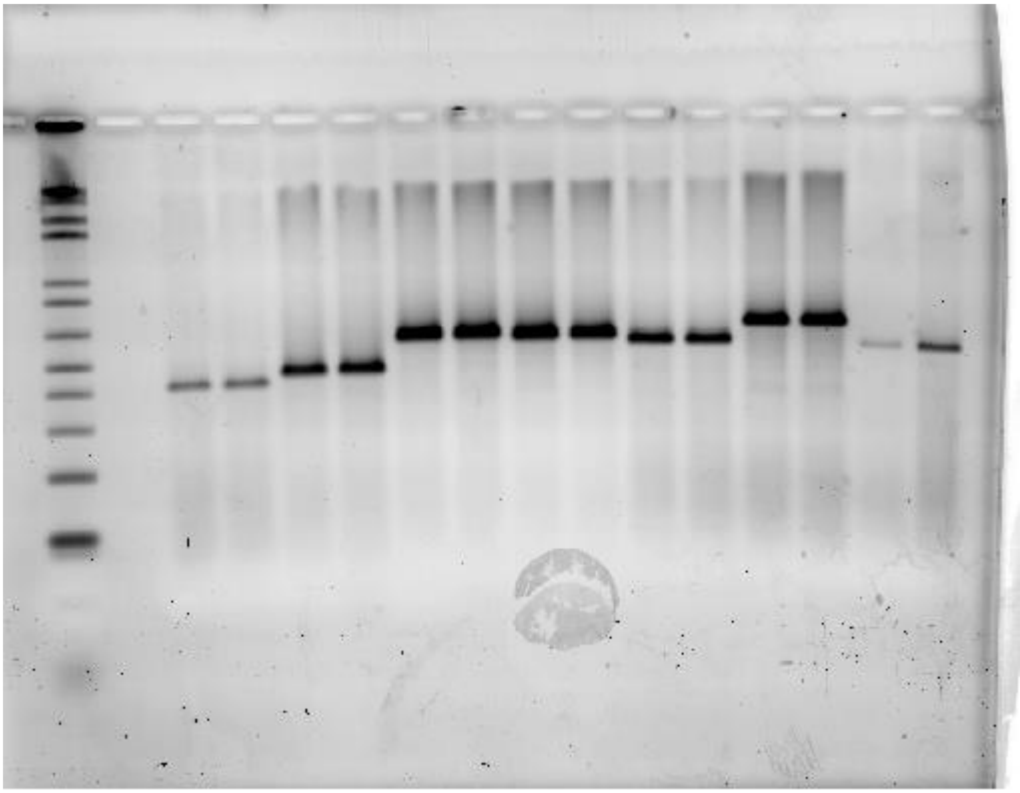

Supplement: Supplementary file 1 — Supplementary Info [file 41598_2017_9268_MOESM1_ESM.pdf]
